# Physics-informed two-tier neural network for non-linear model order reduction

**DOI:** 10.1186/s40323-024-00273-3

**Published:** 2024-11-14

**Authors:** Yankun Hong, Harshit Bansal, Karen Veroy

**Affiliations:** Centre for Analysis, Scientific Computing and Applications, Eindhoven University of Technology, Eindhoven, 5600MB The Netherlands

**Keywords:** Physics-informed machine learning, Neural networks, Non-linear model order reduction, Hyper-reduction

## Abstract

In recent years, machine learning (ML) has had a great impact in the area of non-intrusive, non-linear model order reduction (MOR). However, the offline training phase often still entails high computational costs since it requires numerous, expensive, full-order solutions as the training data. Furthermore, in state-of-the-art methods, neural networks trained by a small amount of training data cannot be expected to generalize sufficiently well, and the training phase often ignores the underlying physical information when it is applied with MOR. Moreover, state-of-the-art MOR techniques that ensure an efficient online stage, such as hyper reduction techniques, are either intrusive or entail high offline computational costs. To resolve these challenges, inspired by recent developments in physics-informed and physics-reinforced neural networks, we propose a non-intrusive, physics-informed, two-tier deep network (TTDN) method. The proposed network, in which the first tier achieves the regression of the unknown quantity of interest and the second tier rebuilds the physical constitutive law between the unknown quantities of interest and derived quantities, is trained using pretraining and semi-supervised learning strategies. To illustrate the efficiency of the proposed approach, we perform numerical experiments on challenging non-linear and non-affine problems, including multi-scale mechanics problems.

## Introduction

Numerical methods, such as the finite element method (FEM) [1], are extensively employed in science, technology, and engineering due to the absence of analytical solutions for the partial differential equations (PDEs) governing various physical, chemical, and other phenomena. However, the computational costs of such numerical simulations are often very high, particularly for large-scale, complex applications. In this paper, we aim to leverage the strengths of two approaches—model order reduction and machine learning—to reduce this high computational cost. We begin by briefly reviewing relevant work in MOR and ML.

Over the past few decades, model order reduction techniques have emerged as a viable solution to address the computational challenges associated with large-scale systems. These techniques, such as the reduced basis method (RBM), proper orthogonal decomposition (POD), balanced truncation, rational interpolation methods, among others, have been proposed to reduce the degrees of freedom in the original full-order system [2]. A comprehensive review of these techniques can be found in, e.g., [2–4]. For widely used methods, such as RBM and POD, dimension reduction is typically achieved by constraining the function space of the unknown quantity to a low-rank subspace that captures the manifold of solutions over a range of parameters [4, 5]. In these methods and in the case of non-affine or non-linear methods, an additional level of reduction has been developed to either construct an approximate affine decomposition or to reduce the cost of integration via hyper-reduction. Approximate affine decomposition or hyper-reduction, when combined with standard MOR techniques, facilitates an offline-online computational decomposition [4]. Approximate affine decomposition is often accomplished using the empirical interpolation method (EIM) [4–7], the discrete empirical interpolation method (DEIM) [8], other variants of EIM [9, 10], or adaptive cross approximation [11–13], etc. In hyper-reduction, some methods, such as the aforementioned EIM-type approaches, gappy-POD [14–18] and its extensions [19, 20], are designed to isolate the terms in the non-linearity that exclusively depend on spatial coordinates. This approach aligns with the concept of affine decomposition, which aims to separate parameters from spatial coordinates. Hence, we regard affine decomposition as a special application of hyper-reduction and refer the techniques for affine decomposition and hyper-reduction as general hyper-reduction techniques. Alternatively, various other hyper-reduction methods based on approximate integration have been developed. These methods include reduced order quadrature [21, 22], reduced integration domain method [23], empirical cubature method [24, 25], as well as energy-conserving sampling and weighting techniques [26, 27], among others. Many of the aforementioned general hyper-reduction methods can be challenging to implement within the finite element (FE) framework, as they often require access to the Jacobian, the intermediate state of the Newton iteration, and so on, and those intrusive terms are often inaccessible in many FEM packages. In addition, constructing those term often demands laborious, problem-specific, and manually-designed codes, which poses challenges.

In recent years, machine learning has emerged as a powerful tool for approximating functions in various applications [28]. As a result, there has been a growing research interest in utilizing ML techniques for (i) solving PDEs and (ii) building inexpensive surrogates of PDE solutions in combination with MOR methods. One approach for using ML to solve PDEs is through physics-informed neural networks (PINNs) [29–31]. PINNs employ a (deep) neural network (NN) to directly approximate the unknown quantity of interest, leveraging the automatic differentiation capabilities of the NN to enforce the residual of the PDE. These techniques have been proposed not only for solving forward problems [30, 31] but also for addressing inverse problems, see, e.g., [32]. PINNs, being a mesh-free method, exhibit excellent performance in high-dimensional problems where traditional discretization approaches face challenges. Another strategy, known as deep operator networks (DeepONets) [33], seek to learn the operators, or functionals, in a fully data-driven way, making use of the excellent ability of neural networks to approximate the non-linearity as a universal approximation. An extension of DeepONets, namely physics-informed DeepONets [34], unify the advantages of the physics-informed strategy from PINNs and the capability of operator learning from DeepONets, and exhibits better consistency to the governing physical systems. However, the training stage of both PINNs and DeepONets is generally more computationally expensive than the FE method. A data-driven improvement of PINNs, leveraging multifidelity data and deep networks to learn upscaling from low fidelity to high fidelity, has been investigated as multifidelity PINNs [35, 36]. Such multifidelity and multi-network strategy has also been applied in operator learning, with DeepONets, see, e.g., [37, 38]. ML techniques have also been used in conjunction with MOR to address specific challenges encountered in standard MOR approaches, such as intrusiveness and non-linearity [39–41]. These hybridized approaches aim to leverage the strengths of both ML and MOR to improve computational efficiency and accuracy in solving PDEs.

The existing hybridized MOR-ML techniques can broadly be classified into two categories. One of these involves the use of neural networks, specifically autoencoders, to directly learn the non-linear reduced basis manifold and achieve dimension reduction [42–44]. This approach has been reported to achieve higher compression rates compared to the standard POD algorithm [45]. Several architectures of autoencoders have been explored for this purpose, and we refer to [46–50] for more details. Additionally, improvements have been proposed by combining autoencoders with the POD algorithm [51]. However, despite the development of such hierarchical schemes, training becomes more challenging and computationally expensive when dealing with larger networks. Moreover, in cases where the geometrical shape of the computational domain is complex or variable, in order to preserve the sparsity of the network, more advanced alternatives to conventional autoencoders such as graph autoencoders may be necessary [52] although they come with increased complexity, both in terms of implementation and computational requirements. Another category, which we pursue in this work, involves generating reduced bases using the POD algorithm and subsequently using ML techniques for constructing a regression map to predict the coefficients of the reduced basis [41, 53]. In the past, various non-intrusive approaches have been proposed for constructing the regression map, including Gaussian process regression or Kriging [39, 54, 55], NN [40, 56, 57], among others. This line of thought has also been extended to handle dynamical systems using recurrent neural networks and long short-term memory models [58–60]. Additionally, a combination of Gaussian process and neural networks, known as neural-net-induced Gaussian process regression, has been introduced in [61]. The theoretical complexity analysis for deep neural networks with respect to the required accuracy, combined with model order reduction, has been discussed in [62, 63] for the ReLU and ReQU activation functions. The aforementioned hybrid MOR-ML methods are non-intrusive, but they often require a large amount of training data to achieve high generalization accuracy. However, generating such training data, which typically consists of full-order solutions, can be extremely expensive, especially in cases where the parameter dimension is high or the parameter-dependence is complex. To address these challenges, researchers have sought methods to enhance generalization capability by improving the training phase, thereby reducing the generalization error with limited full-order training data. The physics-reinforced neural network (PRNN), proposed in [64], attempts to address this issue by incorporating PDE information into the training stage. This physics-informed ML method combines the strategy of PINNs, which minimize the residual of the PDE, with the non-intrusive MOR-ML methods that build regressions based on the full-order solution data. The key idea behind this method is that incorporating the physical laws that govern the PDE, which are not explicitly accounted for in standard MOR-ML methods, can enhance the quality of the regression and improve generalization performance.

Building upon previous works, we propose a two-tier deep network that combines the advantages of physics-informed ML techniques and non-intrusive MOR-ML methods. Our approach aims to incorporate the physical laws into the training phase without sacrificing the non-intrusive nature of MOR-ML methods while improving the generalization capacity under limited full-order training data. To this end, in contrast to the non-intrusive ML method in [40] and the physics-informed ML method in [64], our proposed method utilizes a two-tier (deep) neural network architecture. The first tier consists of a neural network, similar to those used in non-intrusive methods [40, 64], which predicts the unknown quantity of interest. This network serves as a regression model for the quantity of interest as a function of the input parameters. The second tier of the network predicts the derived quantity of interest, which represents the explicit non-linear term appearing in the governing equation(s). Examples of derived quantity of interest include physical quantities determined by constitutive laws, such as stress in mechanics problems or heat flux in thermal problems^1^. In principle, the second tier constructs a reduced-order space for the non-linearity using the POD algorithm. The neural network is then utilized to predict the coefficients, achieving an affine-decomposable approximation of the non-linearity. The goal of the second tier is hence to approximate the constitutive law and improve the generalization accuracy of the first tier. To achieve this, the proposed two-tier deep network is trained using pretraining and semi-supervised learning strategies. By leveraging the information from the underlying physics, which enables semi-supervised learning, numerical tests show that our method reduces the generalization error of the regression model with less full-order data compared to the standard non-intrusive MOR-ML method. The used semi-supervised learning method is similar to the training stage for multifidelity PINNs in [35, 36].

Specifically, the second tier of the network can also be viewed as a non-intrusive general hyper-reduction method since it efficiently captures the possibly nonlinear relationship between the unknown quantity of interest and the governing PDE during the training stage. By leveraging automatic differentiation techniques, the network can access the Jacobian in a straightforward manner, eliminating the need for auxiliary hand-designed and intrusive codes. Note that the application of neural networks to hyper-reduction has also been investigated in previous works such as [56, 65]. In [56], the authors used neural networks to directly predict the residual of the reduced PDE in the MOR framework, while, in [65], this strategy is applied to hyper-reduction using a basis obtained from a Convolutional Autoencoder. Furthermore, PRNN, introduced in [64], which incorporates the original PDE as a part of the loss function, enhances the generalization capability of the network. However, there is still a need to improve the generalization capacity of the NN-based hyper-reduction technique under limited full-order training data. In this work, via the proposed two-tier deep network, we aim to overcome the limitations of existing affine decomposition or hyper-reduction methods, and to handle more complex and non-linear problems, offering an alternative perspective on the integration of model order reduction and physics-informed machine learning techniques.

*Main contributions*: In this work, we present a non-intrusive method via physics-informed ML that combines the principles and strengths of MOR and ML. Our proposed method offers several advantages in scenarios where the full-order training data is limited and expensive. The key features of our method are as follows:

- It enhances the generalization accuracy for both the unknown and derived quantities in a manner consistent with the underlying physics.
- It achieves comparable accuracy to existing methods while requiring less full-order training data, which is typically costly to obtain.
- It can be interpreted as an efficient, non-intrusive approach to general hyper-reduction since it employs neural networks to approximate constitutive laws.

Through the integration of ML-based MOR principles and physics-informed ML, our method addresses the challenges associated with limited training data and offers improved accuracy and efficiency. To demonstrate the efficacy of our approach, we evaluate it alongside existing state-of-the-art methods on a set of challenging non-linear and non-affine problems, including multi-scale mechanics problems. Through these extensive evaluations, we gain insights into the performance and computational trade-offs associated with various approaches, which eventually helps us to highlight the advantages and limitations of our method. Additionally, we investigate the impact of utilizing different orders of finite elements, such as P1 and P2, to compare their behavior in terms of computational costs associated with obtaining the full-order data.

*Outline*: The remainder of the paper is organized as follows. The mathematical description of the problem is provided in Sect. “Model description”. We then introduce the full-order model based on the finite element method in Sect. “Full-order model”. We next discuss the reduced basis method as a MOR technique in Sect. “Reduced basis method”. To address non-affine and non-linear problems in MOR, we introduce general hyper-reduction in Sect. “General hyper-reduction”. Section Two-tier physics-informed deep network outlines our proposed non-intrusive, two-tier, physics-informed ML method. The proposed method, together with the components for training and the detailed training algorithm, is presented in Sect. “Main ingredients of the proposed network”. We further provide an alternative perspective on the proposed method in Sect. “Variant of TTDN”. We discuss the use of this viewpoint as a general hyper-reduction strategy and the connection of our proposed method to existing methods in Sect. “Connection to existing methods”. In Sect. “Numerical experiments”, we conduct several numerical case studies and compare the results obtained using the proposed method with those achieved by the existing state-of-the-art methods. Finally, in Sect. “Conclusions and outlook”, we conclude the paper and discuss potential future directions for research.

## Problem statement

### Model description

Consider a general non-linear, steady, parametrized PDE that reads: Find the state $u({\varvec{{x}}}) \in \hat{H}(\Omega )$, such that

1 $$\begin{aligned} \mathcal {A}_{\varvec{\mu }} P(u({\varvec{{x}}}); \varvec{\mu }) + \tilde{\mathcal {A}}_{\varvec{\mu }} u({\varvec{{x}}}) = s({\varvec{{x}}}), \end{aligned}$$

where $\varvec{\mu }$ is a parameter or parameter vector in the parameter space $\mathbb {M}$, $\hat{H}(\Omega )$ is a Hilbert space, $\Omega$ is the domain of interest, *s* is a source term, and *u* is the unknown state. Here, $P(\cdot ; \cdot )$ is a non-linear function of *u* and is parameterized by $\varvec{\mu }$, and the $\varvec{\mu }$-dependent operators $\mathcal {A}_{\varvec{\mu }}$ and $\tilde{\mathcal {A}}_{\varvec{\mu }}$ are linear in *P* and *u*, respectively. Hence, the non-linearity of the PDE is embedded only in *P* and not in $\mathcal {A}_{\varvec{\mu }}$.

In a physical sense, (1) is a balance equation of the physical quantity *P* that is derived from the unknown state *u*. We will refer to *P* as the derived or equilibrium quantity. For example, in quasi-static mechanics, *u* is the displacement, *P* is the stress, $\mathcal {A}_{\varvec{\mu }} = \nabla \cdot$ is the divergence operator, $\tilde{\mathcal {A}}_{\varvec{\mu }} = 0$, *s* is a body force; in heat transfer, *u* is the temperature, *P* is the heat flux, $\mathcal {A}_{\varvec{\mu }}$ is the divergence operator, $\tilde{\mathcal {A}_{\varvec{\mu }}} = 0$, and *s* is the volumetric heat source.

Without any loss of generality, we omit the $\tilde{\mathcal {A}}_{\varvec{\mu }} u$ term for simplification, since techniques that will be introduced for the term $\mathcal {A}_{\varvec{\mu }} P$ are applicable for $\tilde{\mathcal {A}}_{\varvec{\mu }} u$ as the latter can be considered as a special case of the former by assuming $P(u({\varvec{{x}}}); \varvec{\mu }) = u({\varvec{{x}}})$.

The weak form of (1) can be expressed as: Find $u({\varvec{{x}}}) \in \hat{H}(\Omega )$, such that $\forall v \in \hat{H}(\Omega )$,

2 $$\begin{aligned} \left\langle \mathcal {A}_{\varvec{\mu }} P(u({\varvec{{x}}}); \varvec{\mu }), v\right\rangle = f_{\varvec{\mu }} (v), \end{aligned}$$

where $\left\langle \cdot , \cdot \right\rangle$ is a bilinear form. Here, $f_{\varvec{\mu }}(v) := f_s(v; \varvec{\mu }) + f_b(v; \varvec{\mu })$ is a linear form with respect to *v*, where $f_s(v;\varvec{\mu }) = \left\langle s, v\right\rangle$ is derived from the source term and $f_b(v; \varvec{\mu })$ is a term that accounts for the (imposed) boundary conditions.

The residual of the weak formulation (2) is given by:

3 $$\begin{aligned} \mathcal {R}(P(u; \varvec{\mu }), v; \varvec{\mu }) := f_{\varvec{\mu }}(v) - \left\langle \mathcal {A}_{\varvec{\mu }} P(u({\varvec{{x}}}); \varvec{\mu }), v\right\rangle , \end{aligned}$$

and the objective can now be expressed in the following way: Find $u \in \hat{H}(\Omega )$ such that its derived quantity $P(u; \varvec{\mu })$, which follows the constitutive law (physical information), causes the residual to vanish, i.e., $\mathcal {R}(P(u; \varvec{\mu }), v; \varvec{\mu }) = 0$ for all $v \in \hat{H}(\Omega )$.

In the unsteady case,

4 $$\begin{aligned} \mathcal {A}_{\varvec{\mu }} P(u({\varvec{{x}}}, t); \varvec{\mu }) + \tilde{\mathcal {A}}_{\varvec{\mu }} u({\varvec{{x}}}, t) = \frac{\partial u({\varvec{{x}}}, t)}{\partial t} + s({\varvec{{x}}}), \end{aligned}$$

the use of a time discretization scheme such as the Euler discretization with time step $\delta t$ yields

5 $$\begin{aligned} \left\langle \mathcal {A}_{\varvec{\mu }} P(u({\varvec{{x}}}, t^n); \varvec{\mu }), v\right\rangle - \frac{1}{\delta t} \left\langle u({\varvec{{x}}}, t^n), v\right\rangle = f_{\varvec{\mu }} (v) - \frac{1}{\delta t} \left\langle u({\varvec{{x}}}, t^{n-1}), v\right\rangle , \qquad \forall v \in \hat{H}(\Omega ), \end{aligned}$$

where $u({\varvec{{x}}}, t^n) \in \hat{H}(\Omega )$. We note that equation (5) is still of the form (2) since, for a given time $t^n$, the term $\frac{1}{\delta t} \left\langle u({\varvec{{x}}}, t^n), v\right\rangle$ is of the form $\tilde{\mathcal {A}}_{\varvec{\mu }} u({\varvec{{x}}})$ in (1), and $\left\langle u({\varvec{{x}}}, t^{n-1}), v\right\rangle$ is of the form $f_{\varvec{\mu }} (v)$ in (2). We thus focus our attention on problems of the type (2) and note that this general form also encompasses unsteady problems of the type (4) and (5).

#### Remark 1

The residual $\mathcal {R}$ plays a key role in this work. It plays an essential role in training the neural network that minimizes the squared residual for any input of the neural network. In fact, several physics-informed machine learning methods, employed to solve PDEs, make use of $\mathcal {R}$ as the loss function, see, for example, the methods proposed in [64] and [31].

### Full-order model

We now introduce a finite element approximation to (2). Given a sufficiently rich FE space $\hat{H}^{\textrm{f}} \in \hat{H}(\Omega )$, find $u^{\textrm{f}} \in \hat{H}^{\textrm{f}}$ satisfying

6 $$\begin{aligned} \mathcal {R}(P(u^{\textrm{f}}; \varvec{\mu }), v; \varvec{\mu }) = f_{\varvec{\mu }}(v) - \left\langle \mathcal {A}_{\varvec{\mu }} P(u^{\textrm{f}}; \varvec{\mu }), v\right\rangle = 0, \qquad \forall v \in \hat{H}^{\textrm{f}}, \end{aligned}$$

where the superscript “$\textrm{f}$” stands for *full-order*. We will hence henceforth refer to (6) as our full-order model. For simplicity of notation, we will also omit the $\varvec{\mu }$ dependence in $\mathcal {A}_{\varvec{\mu }}$ and $f_{\varvec{\mu }}$; we will, however, continue to write $P(\cdot ; \varvec{\mu })$ as this will prove useful.

In the general case in which (6) is nonlinear, we employ Newton’s method: at iteration *n*, and with current guess $u_n^\textrm{f}$, we find $\delta u_n^{\textrm{f}}$ satisfying

7 $$\begin{aligned} - \left\langle \mathcal {A} P^\prime (u^{\textrm{f}}_n, \delta u^{\textrm{f}}_n; \varvec{\mu }), v \right\rangle = \mathcal {R}(P(u^{\textrm{f}}_n; \varvec{\mu }), v; \varvec{\mu }) := f(v) - \left\langle \mathcal {A} P(u^{\textrm{f}}_n; \varvec{\mu }), v\right\rangle , \qquad \forall v \in \hat{H}^{\textrm{f}}, \end{aligned}$$

and let $u_{n+1}^{\textrm{f}} \leftarrow u_n^{\textrm{f}} + \delta u_n^{\textrm{f}}$. Here, $P^\prime (u^{\textrm{f}}_n, \delta u_n^{\textrm{f}}; \varvec{\mu })$ is the Gâteaux derivative of $P(\cdot ; \varvec{\mu })$ at $u_n^{\textrm{f}}$ in the direction $\delta u_n^{\textrm{f}} \in \hat{H}^{\textrm{f}}$. Note that the bilinear form $\left\langle \mathcal {A} P^\prime (u_n^{\textrm{f}}, \cdot ; \varvec{\mu }), \cdot \right\rangle$ representes the Jacobian of $\mathcal {R}$ with respect to $u^{\textrm{f}}$ at $u_n^{\textrm{f}}$.

The matrix form of (7) for the generalized coordinate ${\textbf {u}}^{\textrm{FE}}$ corresponding to $u^{\textrm{f}}$, such that $u^{\textrm{f}} \approx \sum _{i = 1}^{N^{\textrm{f}}} ({\textbf {u}}^{\textrm{FE}})_i \phi _i^{\textrm{FE}}$ based on the finite element basis $\{\phi _i^\textrm{FE}\}_{i = 1}^{N^{\textrm{f}}}$, for the *n*-th iteration step, can then be written as

$$\begin{aligned} {\textbf {M}}^{\textrm{FE}}({\textbf {u}}_n^{\textrm{FE}}; \varvec{\mu }) \cdot \delta {\textbf {u}}_n^{\textrm{FE}} = {\textbf {f}}^{\textrm{FE}} - {\textbf {p}}^\textrm{FE}({\textbf {u}}_n^{\textrm{FE}}; \varvec{\mu }), \end{aligned}$$

where the matrix ${\textbf {M}}^{\textrm{FE}} \in \mathbb {R}^{N^{\textrm{f}} \times N^{\textrm{f}}}$, the vectors ${\textbf {f}}^{\textrm{FE}}$ and ${\textbf {p}}^{\textrm{FE}} \in \mathbb {R}^{N^{\textrm{f}}}$, and $N^{\textrm{f}}$ represents the dimension (also, number of degrees of freedom) of the full-order model. For the details of the aforementioned finite element matrix and vectors, please refer to [1], and also see Appendix A.

## Model order reduction

To reduce the high computational costs associated with solving (7) at each iteration step, we employ MOR, which seeks to approximate the full-order model using a low-rank reduced basis. The conventional methods for generating the reduced basis include proper orthogonal decomposition, greedy algorithm, etc., see, e.g., [4, 5, 66, 67]. A greedy algorithm avoids large numbers of full-order solutions for the training stage at the price of a sophisticated a-posteriori error estimator, which is, however, likely inaccessible in the context of some non-linear problems. In view of this difficulty and considering that, in this work, we are interested in non-linear problems, we exploit the POD algorithm, also known as Karhunen-Loève decomposition in the theory of stochastic processes and as principal component analysis in statistics and machine learning community [4, 68], for the reduced basis generation.

### Reduced basis method

To reduce the computational cost of solving the full-order system, (7), we introduce a reduced-order approximation, $u^{\textrm{r}} \approx u$, where $u^{\textrm{r}}$ is in a (low-dimensional) Hilbert space, $\hat{H}^{\textrm{r}}$, that approximates $\hat{H}$, with the superscript “$\textrm{r}$” denoting the reduced-order. Analogous to (7), we then find, at *n*-th Newton step, the increment, $\delta u^{\textrm{r}}_n \in \hat{H}^{\textrm{r}}$, such that, for all $v \in \hat{H}^{\textrm{r}}$,

8 $$\begin{aligned} - \left\langle \mathcal {A} P^\prime (u^{\textrm{r}}_n, \delta u^{\textrm{r}}; \varvec{\mu }), v \right\rangle = \mathcal {R}(P(u_n^{\textrm{r}}; \varvec{\mu }), v; \varvec{\mu }) := f(v) - \left\langle \mathcal {A} P(u_n^{\textrm{r}}; \varvec{\mu }), v\right\rangle . \end{aligned}$$

We now briefly summarize the matrix formulation for (8). Given the basis $\{\phi _i^{\textrm{RB}}\}_{i = 1}^{N^\textrm{r}}$ obtained using, e.g., the POD algorithm (see, e.g., [4, 5]), by $\phi _i^{\textrm{RB}} = \sum _{j=1}^{N^{\textrm{f}}} [{\textbf {B}}]_{i,j} \phi _j^{\textrm{FE}}$, where $N^{\textrm{r}} \ll N^{\textrm{f}}$, for the *n*-th iteration step,

9 $$\begin{aligned} {\textbf {M}}^{\textrm{RB}}({\textbf {u}}_n^{\textrm{RB}}; \varvec{\mu }) \cdot \delta {\textbf {u}}_n^{\textrm{RB}} = {\textbf {f}}^{\textrm{RB}} - {\textbf {p}}^\textrm{RB}({\textbf {u}}_n^{\textrm{RB}}; \varvec{\mu }), \end{aligned}$$

and ${\textbf {u}}^{\textrm{FE}} \approx {\textbf {B}}^T \cdot {\textbf {u}}^{\textrm{RB}}$ holds. The relation between the full-order model and the reduced-order model reads:

10 $$\begin{aligned} {\textbf {M}}^{\textrm{RB}}({\textbf {u}}_n^{\textrm{RB}}; \varvec{\mu })&= {\textbf {B}} \cdot {\textbf {M}}^{\textrm{FE}}({\textbf {B}}^T \cdot {\textbf {u}}_n^{\textrm{RB}}; \varvec{\mu }) \cdot {\textbf {B}}^T; \end{aligned}$$

11 $$\begin{aligned} {\textbf {f}}^{\textrm{RB}}&= {\textbf {B}} \cdot {\textbf {f}}^{\textrm{FE}}; \end{aligned}$$

12 $$\begin{aligned} {\textbf {p}}^{\textrm{RB}}({\textbf {u}}_n^{\textrm{RB}}; \varvec{\mu })&= {\textbf {B}} \cdot {\textbf {p}}^{\textrm{FE}}({\textbf {B}}^T \cdot {\textbf {u}}_n^{\textrm{RB}}; \varvec{\mu }). \end{aligned}$$

The RBM with (10)-(12) is referred to as the projection-based RBM in the following. For more details about the derivation of this reduced model, see [5]. For the definitions of the matrices and vectors, see Appendix A.

The RBM is only effective if the computation can be decomposed into an offline and an online stage; see, e.g., [4, 5], for more details. If the decomposition is possible, in the offline stage, we construct and store the reduced basis, i.e., ${\textbf {B}}$, and all the other parametrically-independent terms, e.g., ${\textbf {f}}^\textrm{RB}$ in (11). In the online stage, the computational cost is independent of the dimension of the full-order model. Only the terms that depend on the parameter of interest need to be computed in this stage. Thus, we can quickly reconstruct and solve (9). However, the existence of non-linear terms (10) and (12) challenges the offline-online decomposition since these terms depend not only on the parameter $\varvec{\mu }$ but also on the iteration state ${\textbf {u}}_n^{\textrm{RB}}$, which can not be computed and stored in advance in the offline stage. Consequently, we have to perform the corresponding computations to reconstruct (9). Given that the reconstruction of (10) and (12) at each iteration step is expensive and related to the full-order $N^{\textrm{f}}$, we next introduce the existing notion of general hyper-reduction to reduce the online costs; see the following subsection.

### General hyper-reduction

Recall that, in (9), we need to compute the vector ${\textbf {p}}^{\textrm{RB}}({\textbf {u}}_n^{\textrm{RB}}; \varvec{\mu }) = {\textbf {B}} \cdot {\textbf {p}}^{\textrm{FE}}({\textbf {B}}^T \cdot {\textbf {u}}_n^{\textrm{RB}}; \varvec{\mu })$ and ${\textbf {M}}^\textrm{RB}({\textbf {u}}_n^{\textrm{RB}}; \varvec{\mu }) = {\textbf {B}} \cdot {\textbf {M}}^{\textrm{FE}}({\textbf {B}}^T \cdot {\textbf {u}}_n^{\textrm{RB}}; \varvec{\mu }) \cdot {\textbf {B}}^T$ in the online stage. The decomposition-based hyper-reduction methods, e.g., EIM [7] and gappy-POD [17], introduce an approximation to the non-linearity. In the context of the problem at hand, this entails approximating *P* by:

13 $$\begin{aligned} P({\textbf {u}}^{\textrm{RB}}; \varvec{\mu })({\varvec{{x}}}) \approx \sum _{p=1}^{Q^{\textrm{r}}} ({\textbf {P}}^{\textrm{HR}}({\textbf {u}}^\textrm{RB}; \varvec{\mu }))_p \xi _p^{\textrm{RB}}({\varvec{{x}}}), \end{aligned}$$

where $Q^{\textrm{r}}$ is the number of hyper-reduction terms, $\{\xi ^{\textrm{RB}}_p({\varvec{{x}}})\}_{p=1}^{Q^{\textrm{r}}}$ is the set of the hyper-reduction terms to approximate the target function *P* in (13), and ${\textbf {P}}^{\textrm{HR}}({\textbf {u}}^{\textrm{RB}}; \varvec{\mu }) \in \mathbb {R}^{Q^{\textrm{r}}}$ is the vector of the hyper-reduction coefficients. In this way, we separate the parameters (here, we also regard ${\textbf {u}}^{\textrm{RB}}$ as parameters) from the spatial coordinates ${\varvec{{x}}}$ and can thus implement the calculations that are related to the spatial coordinates in the offline stage. General hyper-reduction also provides an efficient way to evaluate the map $g^{\textrm{hr}}: (\varvec{\mu }, {\textbf {u}}^{\textrm{RB}}) \mapsto {\textbf {P}}^\textrm{HR}$.

Using (13), we convert the two nonlinear terms (10) and (12) into

14 $$\begin{aligned} {\textbf {p}}^{\textrm{RB}}({\textbf {u}}_n^{\textrm{RB}}; \varvec{\mu })&= {\textbf {B}} \cdot {\textbf {M}}_c \cdot {\textbf {C}}_{\textrm{hr}}^T \cdot {\textbf {P}}^{\textrm{HR}}({\textbf {u}}_n^{\textrm{RB}}, \varvec{\mu }); \end{aligned}$$

15 $$\begin{aligned} {\textbf {M}}^{\textrm{RB}}({\textbf {u}}_n^{\textrm{RB}}; \varvec{\mu })&= {\textbf {B}} \cdot {\textbf {M}}_c \cdot {\textbf {C}}_{\textrm{hr}}^T \cdot {\textbf {J}}^{\textrm{HR}}({\textbf {u}}_n^{\textrm{RB}}, \varvec{\mu }) , \end{aligned}$$

where ${\textbf {M}}_c \in \mathbb {R}^{N^{\textrm{f}} \times Q^{\textrm{f}}}$ is the matrix corresponding to the bilinear form (with respect to *P* and *u*) such that $[{\textbf {M}}_c]_{j,q} = \left\langle \mathcal {A}\xi _q^{\textrm{FE}}, \phi _j^{\textrm{FE}}\right\rangle$ based on the finite element basis $\{\xi _q^{\textrm{FE}}\}_{q = 1}^{Q^{\textrm{f}}}$ for *P* and the basis finite element basis $\{\phi _j^{\textrm{FE}}\}_{j = 1}^{N^{\textrm{f}}}$ for *u*. ${\textbf {C}}_{\textrm{hr}} \in \mathbb {R}^{Q^\textrm{r} \times Q^{\textrm{f}}}$ is the hyper-reduction matrix. Here, the matrix ${\textbf {J}}^{\textrm{HR}} \in \mathbb {R}^{Q^{\textrm{r}} \times N^\textrm{r}}$ is the Jacobian matrix of ${\textbf {P}}^{\textrm{HR}}({\textbf {u}}^\textrm{RB}, \varvec{\mu })$ with respect to ${\textbf {u}}^{\textrm{RB}}$ at ${\textbf {u}}_n^{\textrm{RB}}$. The definitions of the aforementioned matrices, including ${\textbf {M}}_c$, ${\textbf {C}}_\textrm{hr}$ and ${\textbf {J}}^{\textrm{HR}}$, are provided in Appendix A. As a result of the decomposition, governed by (13)–(15), it is feasible to precompute ${\textbf {M}}_c$ and ${\textbf {C}}_{\textrm{hr}}$ in the offline stage, and to evaluate ${\textbf {P}}^{\textrm{RB}}$ and ${\textbf {J}}^{\textrm{RB}}$, which are only dependent on $N^{\textrm{r}}$ and $Q^{\textrm{r}}$, respectively, in the online stage. In this way, we achieve a complete offline-online separation such that the online computation is independent of the dimension of the full-order model.

Although, in the explanation above, we refer only to nonlinear problems, a similar approximation can be employed for non-affine problems (i.e., ones in which the operator does not permit an affine decomposition); we refer the reader to Chapter 10 in [4]. For more details about the application of general hyper-reduction in MOR, we refer the reader to Chapter 11 in [4].

## Two-tier physics-informed deep network

The RBM, in conjunction with general hyper-reduction techniques, provides a good approximation to the full-order model and dramatically reduces the computational cost while keeping high accuracy by achieving an offline-online decomposition. However, in the online stage, non-linear problems still need Newton iterations, which rely on the computation of the Jacobian and (sometimes highly) intrusive hand-designed coding depending on the form of the governing PDE. As discussed in Sect. “Introduction”, the introduction of machine learning, in conjunction with MOR, in order to solve PDEs, signals the importance of non-intrusive, implementation-friendly, and efficient methods. In some methods, the prediction can be faster than the time it takes to assemble and solve the smaller (projection-based) non-linear reduced problem. With this in mind, in this section, we (1) present the proposed physics-informed deep learning method as a novel, non-intrusive ML-based MOR method, (2) show the differences from the existing non-intrusive ML-based MOR methods, e.g., [40, 64], and (3) demonstrate how the proposed method can be used for the non-intrusive affine decomposition and hyper-reduction.

### Main ingredients of the proposed network

Our proposed method, referred to as the TTDN method, uses ingredients from the field of ML and MOR. It shares the idea of many popular non-intrusive ML-based MOR methods, e.g., [40, 53, 64], to construct the reduced basis space $\hat{H}^{\textrm{r}}$ for the unknown quantity of interest *u* by the POD algorithm and, subsequently, employ the regression method to find the coefficients ${\textbf {u}}^{\textrm{RB}}$ of the reduced basis. However, the proposed method differs from other methods in the way it takes the PDE into account while training the network to improve the generalization ability with the limited full-order training data. Next, we present the four necessary components of the proposed neural network: (i) the architecture of the network, (ii) the loss function, (iii) the training data, and (iv) the optimization method, and meanwhile discuss how it can improve the generalization ability in this subsection.

#### The architecture of TTDN

**Fig. 1:**
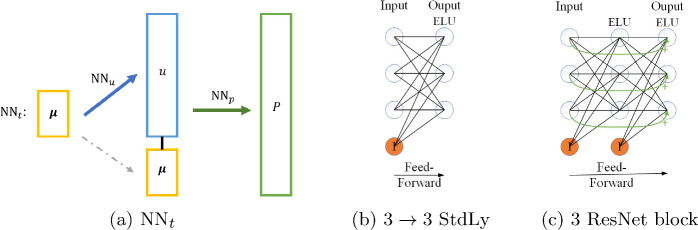
**a** Diagrams of $\textrm{NN}_t$ combined by $\textrm{NN}_u$ and $\textrm{NN}_p$; **b** a layer of the standard network with input width 3 and output width 3; **c** a layer of a ResNet block with width 3

The TTDN is constructed by two tiers of feed-forward neural network with each part realizing its own functionality. The first tier is a feed-forward network that achieves the regression $g_u: \varvec{\mu } \mapsto {\textbf {u}}^{\textrm{RB}}$. We refer this network as $\textrm{NN}_u$. The second tier is another feed-forward network, referred to as $\textrm{NN}_p$, that achieves the regression of $g_p: (\varvec{\mu }, {\textbf {u}}^{\textrm{RB}}) \mapsto {\textbf {P}}^\textrm{RB}$, which is an approximation that represents the underlying physics or, more precisely, the constitutive law between *u* and *P*. Here, ${\textbf {u}}^{\textrm{RB}}$ and ${\textbf {P}}^{\textrm{RB}}$ are the reduced coefficients that approximate the corresponding quantities by, e.g., projecting into the reduced basis space. Finally, we get the structure of the entire TTDN, which is referred to as ${\textrm{NN}}_t$, by the following map ; see the schematic diagram in Fig. 1a.

Next, we describe the detailed architecture of the network $\textrm{NN}_u$ and $\textrm{NN}_p$. The general feed-forward neural network is constructed by one input layer, one output layer, and multiple (denoted by $M_L$) hidden layers, i.e., $(M_L+2)$ layers in total. As a fully connected, feed-forward network, the message can only pass from one layer to the forward layer. The number of neurons of the input layer is $\dim {(\varvec{\mu })}$ for $\textrm{NN}_u$ and $N^{\textrm{r}} + \dim {(\varvec{\mu })}$ for $\textrm{NN}_p$, and for the output layer, the number of neurons is $N^{\textrm{r}}$ for $\textrm{NN}_u$ and $Q^{\textrm{r}}$ for $\textrm{NN}_p$. In general, the hidden layers are constructed by the standard layer with the same structure of the output layer and an additional nonlinear activation function. A possible activation function, which is also used in this work unless otherwise mentioned, is the $\textrm{ELU}$ function that is defined by

$$\begin{aligned} \textrm{ELU}(x) = \left\{ \begin{array} {l l} x, &  x > 0; \\ \exp {(x)} - 1 &  x \le 0, \end{array} \right. \end{aligned}$$

since it shares the efficiency of the activation function $\textrm{ReLU}$ but is differential.

Considering the input with dimension $M_{\textrm{i}}$ and the output with dimension $M_{\textrm{o}}$, the function of a standard $M_{\textrm{i}} \rightarrow M_{\textrm{o}}$ layer with the activation is defined by $\textrm{ELU}({\textbf {W}} \cdot {\varvec{{x}}} + {\varvec{{b}}})$, where we abuse the notation of $\textrm{ELU}(\cdot )$ for a vector argument by applying the scalar functions to the vector element-wise. Here ${\varvec{{x}}} \in \mathbb {R}^{M_{\textrm{i}}}$ is the input argument, and the weight matrix ${\textbf {W}} \in \mathbb {R}^{M_{\textrm{o}} \times M_{\textrm{i}}}$ and the bias vector ${\varvec{{b}}} \in \mathbb {R}^{M_{\textrm{o}}}$ are the training parameters to be optimized. We show a diagram of the standard layer in Fig. 1b, wherein the inputs and the outputs have the same dimension $M_{\textrm{i}} = M_{\textrm{o}} = 3$, and the nodes in the output layer compose the application of the activation function, and henceforth it is denoted as “$3 \rightarrow 3$ StdLy”. Similarly, an input or output layer is denoted as “$M_i$ StdLy” or “$M_o$ StdLy”.

The hidden layer can also be constructed by the residual neural network (ResNet) block, which was first proposed for convolution neural networks [69] and is widely used in very deep convolutional networks for image processing. It is a layer constructed by two standard layers, and the inputs are added as an addendum to the output. Hence, for each ResNet block, the dimension of the inputs should be equal to the dimension of the outputs. More specifically, the function of the $M_{\textrm{i}} \rightarrow M_{\textrm{o}}$ ResNet block is defined by $\textrm{ELU}({\textbf {W}}_2 \cdot \textrm{ELU}({\textbf {W}}_1 \cdot {\varvec{{x}}} + {\varvec{{b}}}_1) + {\varvec{{b}}}_2) + {\varvec{{x}}}$, where ${\varvec{{x}}} \in \mathbb {R}^{M_{\textrm{i}}}$ is the input argument, ${\textbf {W}}_1, {\textbf {W}}_2 \in \mathbb {R}^{M_{\textrm{i}} \times M_{\textrm{i}}}$ and ${\varvec{{b}}}_1, {\varvec{{b}}}_2 \in \mathbb {R}^{M_{\textrm{i}}}$ are the weights and biases of the first and the second layer, respectively. Compared to the standard layer, ResNet has the capacity to skip the connection between layers by assigning very small values to ${\textbf {W}}_1$ and ${\textbf {W}}_2$ and, hence, it can avoid the problem of vanishing or exploding gradients, especially for a deep network. A diagram of the ResNet block with the dimension of inputs (and outputs) $M_{\textrm{i}} = 3$ is shown in Fig. 1c. This block is denoted as “3 ResNet” block. It is worth noting that, in this work, the network is deep since the entire TTDN is constructed by two tiers of network and, hence, the ResNet block is useful.

#### The loss function and the TTDN training

Having already introduced the architecture above, we next discuss the loss function, the required training data, and the training phase for the TTDN method. Essentially, $\textrm{NN}_t$ predicts the RB coefficients ${\textbf {P}}^{\textrm{RB}}$ of the derived quantity *P*. Hence, in line with (3), it seeks to minimize the norm of the residual in the reduced basis scheme, expressed as,

16 $$\begin{aligned} 0 = {\textbf {R}}({\textbf {P}}^{\textrm{RB}}) := \begin{bmatrix} \mathcal {R}(P_{\textrm{rb}}({\varvec{{x}}}), \phi _1^{\textrm{RB}})&\ldots&\mathcal {R}(P_{\textrm{rb}}({\varvec{{x}}}), \phi _N^{\textrm{RB}}) \end{bmatrix}^{T} = {\textbf {f}}^{\textrm{RB}} - {\textbf {L}} \cdot {\textbf {P}}^{\textrm{RB}}, \end{aligned}$$

where $P_{\textrm{rb}}$ denotes the derived quantity corresponding to the coefficient vector ${\textbf {P}}^{\textrm{RB}}$, and ${\textbf {L}} \in \mathbb {R}^{N^{\textrm{RB}} \times Q^{\textrm{RB}}}$ is defined by $[{\textbf {L}}]_{i, p} = \left( \mathcal {A}(\xi _p^{\textrm{RB}}), \phi _i^{\textrm{RB}}\right)$, which can be precomputed and stored in the offline stage. The square of the residual, $\Vert {\textbf {R}}({\textbf {P}}^{\textrm{RB}}(\cdot ))\Vert ^2$, naturally serves as the loss function for $\textrm{NN}_t$. Given that ${\textbf {P}}^{\textrm{RB}} \approx g_{t}(\varvec{\mu })$, this loss function is formulated in terms of the trainable parameters of $g_{t}$ and the training data set $\Xi _{M} = \{\varvec{\mu }^\textrm{train}_m\}_{m=1}^{M_{M}}$ (with the size of $M_{M}$) as follows:

$$\begin{aligned} \mathcal {L}_{M}(g_{t}, \Xi _{M}) = \textrm{MSE}({\textbf {R}} ( g_{t}(\varvec{\mu }^{\textrm{train}}_m)), 0) := \frac{1}{M_{M}} \sum _{m=1}^{M_{M}} \Vert {\textbf {R}} (g_{t}(\varvec{\mu }^{\textrm{train}}_m))\Vert ^2. \end{aligned}$$

Note that the subscript “*M*” in $\Xi _M$ comes from the fact that the dataset contains only the input feature $\varvec{\mu }$.

This loss function is associated to unsupervised learning, as it requires only the input features $\varvec{\mu }^{\textrm{train}}$, avoiding the need for generating training labels via the computationally expensive full-order solver. Consequently, this approach allows for the utilization of an exceptionally large training data size, denoted $M_{M}$, with the training dataset $\Xi _{M}$, to enhance the generalization accuracy.

However, in the TTDN method, before the unsupervised learning, we advocate for the use of a supervised learning approach to pretrain the two tiers, $\textrm{NN}_u$ and $\textrm{NN}_p$, to ensure that they serve as effective approximations for the true mappings $\varvec{\mu } \mapsto {\textbf {u}}^{\textrm{RB}}$ and $(\varvec{\mu }, {\textbf {u}}^{\textrm{RB}}) \mapsto {\textbf {P}}^\textrm{RB}$, respectively. The pretraining of the first tier, $\textrm{NN}_u$, utilizes full-order training data $\Xi _U = \{(\varvec{\mu }_m^{\textrm{train}}, {\textbf {u}}_m^\textrm{train})\}_{m=1}^{M_U}$, where $M_U$ is the size of the dataset, with $\varvec{\mu }^{\textrm{train}}_{\ \bullet }$ and ${\textbf {u}}^\textrm{train}_{\ \bullet }$ representing the input features and output labels, respectively. The corresponding mean-squared error (MSE) loss function for this pretraining stage is defined as

17 $$\begin{aligned} \mathcal {L}_U(g_u, \Xi _U) = \textrm{MSE}(g_u(\varvec{\mu }^\textrm{train}), {\textbf {u}}^{\textrm{train}}) := \frac{1}{M_U} \sum _{m=1}^{M_U} \Vert g_u(\varvec{\mu }^{\textrm{train}}_m) - {\textbf {u}}^{\textrm{train}}_m \Vert ^2. \end{aligned}$$

For the pretraining implementation, we initially generate the full-order training dataset $\Xi _U$ using an FE solver, followed by employing (17) as the loss function to conduct the supervised training of $\textrm{NN}_u$. This initial pretraining phase for $\textrm{NN}_u$ aligns with the projection-driven neural network (PDNN), a conventional ML-MOR method discussed in [40, 64]. In other words, we apply the PDNN method as a pretraining stage for the first tier sub-network in the proposed TTDN method. Unlike PDNN, where high generalization accuracy is imperative, the pretraining phase for $\textrm{NN}_u$ does not necessitate the same degree of accuracy, allowing for a smaller training data size compared to what the PDNN method would typically require.

Unlike the first tier network $\textrm{NN}_u$ that directly captures the parameter-solution map *on* the solution manifold, the second tier network $\textrm{NN}_p$ aims to approximate the constitutive law both *on* and *around* the solution manifold. Consequently, there are some subtle differences compared to the pretraining of $\textrm{NN}_u$. The network $\textrm{NN}_p$, as a map $g_p: (\varvec{\mu }_m, {\textbf {v}}^\textrm{RB}_m) \mapsto {\textbf {P}}^\textrm{RB}$, is designed not only to accurately predict the output for inputs *on* the solution manifold $(\varvec{\mu }_m, {\textbf {u}}^\textrm{RB}_m(\varvec{\mu }_m))$—where ${\textbf {u}}^\textrm{RB}_m(\varvec{\mu }_m)$ is the reduced solution corresponding to $\varvec{\mu }_m$—but also for those inputs $(\varvec{\mu }_m, {\textbf {v}}^\textrm{RB}_m)$ *around* the solution manifold, with ${\textbf {v}}^\textrm{RB}_m \ne {\textbf {u}}^\textrm{RB}(\varvec{\mu }_m)$. $\textrm{NN}_p$ is functionally required to yield the ${\textbf {P}}^\textrm{RB}$ that vanishes the PDE residual if we pass the pair $({\textbf {u}}^\textrm{RB}_m, \varvec{\mu }_m)$, and to yield the output that does not vanish the PDE residual if we pass the pair $({\textbf {v}}^\textrm{RB}_m, \varvec{\mu }_m)$. To meet the latter requirement, $\textrm{NN}_p$ need to be able to accurately predict the ${\textbf {P}}^\textrm{RB} (\varvec{\mu }_m, {\textbf {v}}^\textrm{RB}_m)$ for ${\textbf {v}}^\textrm{RB}_m$ *around* the solution manifold. Such $\textrm{NN}_p$, combined with $\textrm{NN}_u$, allows unsupervised learning, which aims to yield good predictive ${\textbf {u}}^\textrm{RB}_m$ that minimizes the square of the PDE residual.

For this reason, training $\textrm{NN}_p$ necessitates data *around* the solution manifold, which enable $\textrm{NN}_p$ to accurately predict the in-equilibrium quantity ${\textbf {P}}^{\textrm{RB}}$ corresponding to inconsistent $(\varvec{\mu }_m, {\textbf {v}}^{\textrm{RB}}_m)$. The training dataset $\Xi _P$, with size $M_P$ and referred to as the constitutive data, is then generated by the map of the constitutive law

18 $$\begin{aligned} {\textbf {P}}^{\textrm{RB}}({\textbf {v}}^{\textrm{RB}}, \varvec{\mu }) = {\textbf {C}}^T \cdot \mathcal {P}_{\textrm{FE}} (P({\textbf {B}}^T\cdot {\textbf {v}}^{\textrm{RB}}, \varvec{\mu })), \end{aligned}$$

where $\mathcal {P}_{\textrm{FE}}(\cdot )$ denotes the projection operator into $\hat{H}^{\textrm{f}}$. The “around-the-solution-manifold” constitutive data is generated through the following three steps: (i) sampling a broad range of parameters $\varvec{\mu }$ within $\mathbb {M}$, (ii) employing the pretrained network $\textrm{NN}_u$ to predict ${\textbf {v}}^{\textrm{RB}}$ for these parameter samples, and (iii) computing the training labels ${\textbf {P}}^{\textrm{RB}}$ for all training points using (18).

##### Remark 2

Please note that in step (ii), we denote ${\textbf {v }}^{\textrm{RB}}$ rather than ${\textbf {u }}^\textrm{RB}$, acknowledging that $\textrm{NN}_u$, due to its preliminary pretraining, may not accurately predict ${\textbf {u }}^\textrm{RB}$. Consequently, the pairs $({\textbf {v }}^{\textrm{RB}}, \varvec{\mu })$ are considered to be around the solution manifold.

The computational cost for generating one data point $(\varvec{\mu }, {\textbf {v}}^{\textrm{RB}}, {\textbf {P}}^{\textrm{RB}})$, as per (18), is $\mathcal {O}(Q^{\textrm{r}} N^{\textrm{f}} + N^{\textrm{r}}N^{\textrm{f}})$, which depends on the full-order $N^{\textrm{f}}$. As it is the additional computational cost compared to the PDNN method, the efficiency of the proposed TTDN method is based on the assumption that this computation, $\mathcal {O}(Q^{\textrm{r}} N^{\textrm{f}} + N^{\textrm{r}}N^{\textrm{f}})$, is much cheaper than the computation of the full-order training data, which is $\mathcal {O}((N^{\textrm{f}})^3 N^{\textrm{it}})$ using dense linear system solver^2^. In the limit, as $N^{\textrm{f}}$ tends to a large value, the former is orders cheaper than the latter. Intuitively, the computation for the latter needs not only the computation of the former but also its Jacobian and solution to a linear system at every Newton iteration. Hence, it is likely that the full-order solution will be much more expensive than the evaluation of the constitutive law via a full-order FE evaluation. This thus allows us to generate much more auxiliary training data than $\Xi _U$. Augmenting the auxiliary (inconsistent) training data with the one obtained by full-order solutions, we get a larger training dataset, which is denoted as $\Xi _P$ with size $M_P$, where $M_U \ll M_P \ll M_{M}$. Once the training data $\Xi _P$ is generated, we use the same strategy employed for $\textrm{NN}_u$ to pretrain $\textrm{NN}_p$ with the MSE loss function analogous to (17) (we give the new symbol as $\mathcal {L}_P$) and the data $\Xi _P$ in a supervised manner. Finally, we use the obtained training parameters of $\textrm{NN}_p$ to initialize the second tier of $\textrm{NN}_t$.

We now discuss the loss function and training data in the context of training the entire $\textrm{NN}_t$. The loss function is given by

$$\begin{aligned} \begin{aligned} \mathcal {L}_{T}(g_{t}) =&\mathcal {L}_{U}(g_u, \Xi _U) + \mathcal {L}_{P}(g_p, \Xi _P) + \mathcal {L}_{M}(g_{t}, \Xi _{M}) \\ =&\frac{1}{M_U} \sum _{(\varvec{\mu }^{\textrm{t}}, {\textbf {u}}^{\textrm{t}}) \in \Xi _U} \Vert g_u(\varvec{\mu }^{\textrm{t}}) - {\textbf {u}}^{\textrm{t}} \Vert ^2 + \frac{1}{M_P} \sum _{(\varvec{\mu }^{\textrm{t}}, {\textbf {v}}^{\textrm{t}}, {\textbf {P}}^{\textrm{t}}) \in \Xi _P} \Vert g_p(\varvec{\mu }^{\textrm{t}}, {\textbf {v}}^{\textrm{t}}) - {\textbf {P}}^{\textrm{t}} \Vert ^2 \\&+ \frac{1}{M_{M}} \sum _{\varvec{\mu }^{\textrm{t}} \in \Xi _{M}} \Vert {\textbf {R}} (g_{t}(\varvec{\mu }^{\textrm{t}}))\Vert ^2. \end{aligned} \end{aligned}$$

The inclusion of the first 2 terms ($\mathcal {L}_{U}$ and $\mathcal {L}_{P}$) ensures that the information from the full-order model and constitutive law (contained in the training data $\Xi _U$ and $\Xi _P$) are retained and accounted for. It leads to the strategy of semi-supervised learning, which is the hybridization of supervised and unsupervised learning. Hence, the training data for the semi-supervised learning strategy contains $\Xi _U$ and $\Xi _P$, which are used for supervised learning, as well as $\Xi _{M}$ that is used for unsupervised learning.

Through the semi-supervised learning approach with abundant and cost-effective $\Xi _P$ and $\Xi _{M}$ data, we can leverage deeper and more powerful networks for the TTDN while mitigating the risk of overfitting. This presents an advantage compared to PDNN, which typically relies on shallow networks due to the scarcity of available training data. The final required element is the optimizer to minimize the loss function with respect to the trainable parameters of the network. To this end, in this work, we use the Adam optimizer [70]. Having discussed all the necessary ingredients, we show a pseudocode of the training algorithm for the proposed TTDN method in Algorithm 1.

**Algorithm 1:**
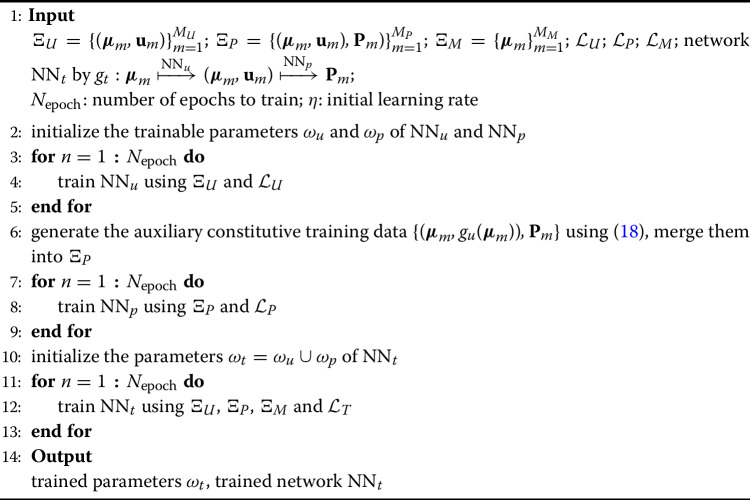
TTDN training

### Variant of TTDN

In this subsection, we show a variant of the proposed TTDN method, which can be helpful to further understand TTDN from another perspective.

The network $\textrm{NN}_u$ has the same functionality as that of the PDNN that was proposed in [40]. In particular, for PDNN, the network serves to predict the coefficients of the unknown quantity *u*. In our TTDN method, the first tier network $\textrm{NN}_u$ also serves to predict the coefficients of *u*. The difference lies in the usage of a different loss function and different training data to train the network. With this in mind, we introduce an alternative variant of the proposed original version of the TTDN method described in previous subsection. In this variant, we will regard $\textrm{NN}_u$ as the only body of the network, and the $\textrm{NN}_p$, together with the residual operator $\mathcal {R}$, only serves as an additional part of the loss that assists to improve the accuracy of $\textrm{NN}_u$ to approximate the unknown quantity. In this view, we have two options to train TTDN: (i) allowing the parameters of the second tier network $\textrm{NN}_p$ to be trainable (this is the option we described in previous subsection and presented in Algorithm 1), and (ii) freezing the parameters of pretrained $\textrm{NN}_p$ while training. For option (ii), the goal is then to train the network $\textrm{NN}_u$ using the loss function defined as

19 $$\begin{aligned} \begin{aligned} \mathcal {L}_{\textrm{PRNN}} (g_u) =&\mathcal {L}_U(g_u, \Xi _U) + \mathcal {L}_{M}(g_p \circ g_u, \Xi _{M}) \\ :=&\frac{1}{M_U} \sum _{(\varvec{\mu }^{\textrm{t}}, {\textbf {u}}^{\textrm{t}}) \in \Xi _U} \Vert g_u(\varvec{\mu }^{\textrm{t}}) - {\textbf {u}}^{\textrm{t}} \Vert ^2 + \frac{1}{M_{M}} \sum _{\varvec{\mu }^{\textrm{t}} \in \Xi _{M}} \Vert {\textbf {R}} (g_p(g_u(\varvec{\mu }^{\textrm{t}}), \varvec{\mu }^{\textrm{t}}))\Vert ^2. \end{aligned} \end{aligned}$$

It is clear that this loss function to learn the unknown quantity *u* is constituted of 2 parts. The first one is the part associated with supervised learning, which applies a data-driven method to learn the approximation. The second one is part of a physics-informed strategy, which makes use of the PDE constraints to enable unsupervised learning to improve the generalization accuracy.

Compared to the second training option, we can see that the first option allows adaptation of $\textrm{NN}_p$ in the semi-supervised learning stage, providing more flexibility. However, the drawback is that it will change the pretrained $\textrm{NN}_p$, which may reduce the accuracy of $\textrm{NN}_p$. Hence, the second training option is useful when we have rich constitutive training data $\Xi _P$. In this case, the pretrained $\textrm{NN}_p$ can achieve very high predictive accuracy, which is effective to improve the accuracy of *u* in the semi-supervised learning. However, in the case when we do not have such a good pretrained $\textrm{NN}_p$, we will select the first option, which allows us to further train $\textrm{NN}_p$ in the semi-supervised learning stage to improve the accuracy of *P*.

### Connection to existing methods

In this subsection, we discuss details about the relation of the proposed TTDN method to some existing non-intrusive ML-based MOR methods such as PRNN [64], PINNs [31] and PDNN [40].

The variant of TTDN exhibits great similarity to PINNs [31]. The loss function of PINNs generally consists of two parts: one part is the MSE between the prediction and the given data (e.g., the boundary value), and the other is the residual of the governing PDE. It shares the same formulation of (19). However, in our proposed method, rather than directly learning and approximating the value of the unknown quantity at the spatial coordinates, we learn the coefficient of the reduced basis of the whole quantity field, thus allowing us to reconstruct the whole unknown quantity field via one single prediction. In addition, in our method, unlike in PINNs, the loss term that corresponds to the PDE residual is approximated by another neural network $\textrm{NN}_p$.

Following again option (ii), we next clarify the relation between the proposed method and PRNN in [64]. PRNN improves on PDNN by adding a fixed residual operator that depends on the underlying PDE with the unknown *u*. This addition to the loss function of the PDNN method serves to enhance the generalization capability of the network. This method, hence, shares the same idea as that of our proposed method. However, our method reconstructs the residual operator of the PDE using the second tier of network—$\textrm{NN}_p$—that enhances the ability of the method to deal with complex non-linear problems. In addition, the true version of TTDN, following the option *(i)*, also enhances the capacity of the network by allowing the parameters of $\textrm{NN}_p$, which appear implicitly in $\text {Residual}(f_u, \Xi _{M})$ in (19), to be trained.

Pursuing this line of thought, we can also find that the role of the second tier network $\textrm{NN}_p$ is, in fact, general hyper-reduction: it performs the regression of $g_p: (\varvec{\mu }, {\textbf {u}}^{\textrm{RB}}) \mapsto {\textbf {P}}^{\textrm{RB}}$ to approximate *P* by

$$\begin{aligned} P({\textbf {u}}^{\textrm{RB}}; \varvec{\mu })({\varvec{{x}}}) \approx \sum _{p=1}^{Q^{\textrm{r}}} ({\textbf {P}}^\textrm{RB}(\varvec{\mu }, {\textbf {u}}^{\textrm{RB}}))_p \xi _p^\textrm{RB}({\varvec{{x}}}), \end{aligned}$$

in the residual operator ${\textbf {R}}$ in (16). In fact, we can regard the reduced basis of *P*, i.e., $\xi _p^{\textrm{RB}}({\varvec{{x}}})$, as the hyper-reduction terms $\xi _p^{\textrm{HR}}({\varvec{{x}}})$ in (13), where as ${\textbf {P}}^{\textrm{RB}}$, which is the output of the second tier $\textrm{NN}_p$, can be considered as the hyper-reduction coefficients. Hence, in this way, the hyper-reduction terms are obtained by the POD algorithm and the hyper-reduction coefficients are predicted by the neural network. This inspires us to use $\textrm{NN}_p$ solely as a general hyper-reduction method.

The advantages of such a method for general hyper-reduction come from the backpropagation algorithm for the derivative and the technique’s non-intrusiveness: the Jacobian matrix ${\textbf {J}}^\textrm{HR} = \frac{\partial {\textbf {P}}^{\textrm{RB}}}{\partial {\textbf {u}}^\textrm{RB}}$ can be computed via efficient automatic differentiation, and the implementation no longer needs problem-specific, and manually-designed codes likes other methods, e.g., EIM.

In fact, such a hyper-reduction technique shares an idea similar to that of the method in [65], which successfully applied neural networks to achieve hyper-reduction, in which basis were obtained using a conventional autoencoder. In this existing method, the RB coefficients, which can also be perceived as the latent variables by conventional autoencoder, are learned, whereas in our method, the basis is obtained via the POD algorithm. In fact, in our work, to obtain an efficient residual operator ${\textbf {R}}(\cdot )$ in (16) such that it can be used as the loss function, we require a known and computationally-efficient map of the residual ${\textbf {R}}$ that allows to evaluate the norm given the reduced-order coefficients of *P*. Recall that the map of the residual in these latent variables is unknown and non-linear, and that the POD algorithm achieves a linear decomposition and meets the aforementioned requirement. Hence, for applying TTDN to several problems considered in the scope of this work, in contrast to [65], we apply the POD algorithm to generate the basis of *P*.

## Numerical experiments

In this section, we implement the proposed method and discuss its performance in several numerical experiments. In Sect. Experiments for affine decomposition and hyper-reduction, we show some brief examples where the second tier network, $\textrm{NN}_p$, can be interpreted as a general hyper-reduction method within the context of MOR. To this end, the numerical case studies include a non-linear thermal problem (Subsect. “Non-linear heat transfer problem”), a linear non-affine problem (Subsect. “Non-affine heat transfer problem”), and a time-dependent Burgers’ problem (Subsect. “Time dependent Burgers’ problem”). In Sect. Experiments for TTDN, we turn to the main method of this work, i.e., the TTDN method, and apply it to two non-linear problems: a non-linear heat transfer problem (Subsect. “Non-linear heat transfer problem”), and a multi-scale hyper-elasticity problem (Subsect. “Hyper-elasticity problem in multi-scale framework”).

In this work, all the experiments are implemented in Python using a docker image running in the following environment: Ubuntu 20.04.4 LTS by Windows Subsystem for Linux (WSL2) in a Windows 10 laptop with CORE i7 6C12T CPU and 32GB RAM, and no GPU accelerators. The geometry of the computational domain and the mesh are created using the open-source software GMSH [71]. The full-order FE solutions are computed using the package FEniCS [72, 73], and the neural network training and prediction phase are implemented using the package PyTorch [74]. The figures are plotted using several packages, namely Matplotlib [75], GMSH and ParaView [76]. The other notable packages used in this work include NumPy [77] and MPI4Py [78]. The parallel computation via MPI4Py is used for generating the training data. This includes the generation of the training dataset for POD, neural network, and EIM, i.e., generating $\Xi _U$ via full-order FE solver and generating $\Xi _P$ via evaluating the constitutive law. Precisely, each data point ($(\varvec{\mu }_m, {\textbf {u}}^\textrm{RB}_m) \in \Xi _U$ or $(\varvec{\mu }_m, {\textbf {v}}^{\textrm{RB}}_m, {\textbf {P}}^{\textrm{RB}}_m) \in \Xi _P$) is computed in parallel via MPI4Py.

### Experiments for affine decomposition and hyper-reduction

In this subsection, we show how the second tier sub-network, $\textrm{NN}_p$, in conjunction with the reduced basis method, works as a tool for affine decomposition or hyper-reduction, and discuss its efficiency by comparing it with state-of-the-art methods.

#### Non-linear heat transfer problem

**Fig. 2:**
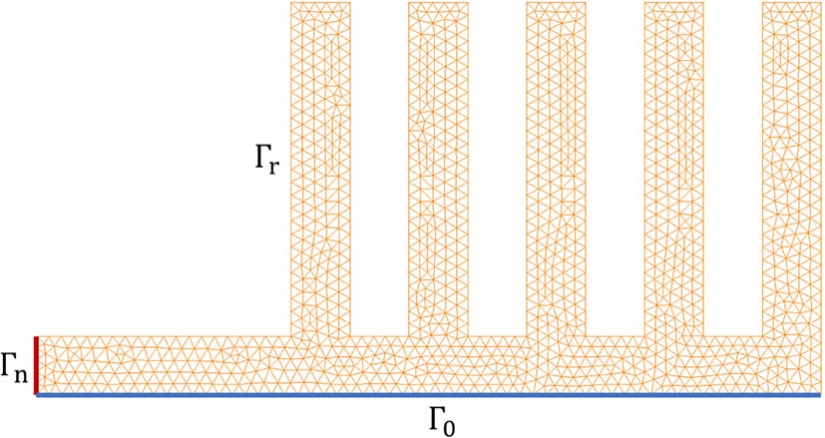
The domain $\Omega$ and the mesh

*Problem description:* We consider a (non-linear) thermal problem governed by:

20 $$\begin{aligned}&- \nabla \cdot {\varvec{{q}}}({\varvec{{x}}}) = 0, \quad {\varvec{{x}}} \in \Omega ; \end{aligned}$$

21 $$\begin{aligned}&{\varvec{{q}}} = - \kappa (u, \varvec{\mu }) \nabla u({\varvec{{x}}}), \quad {\varvec{{x}}} \in \Omega ; \end{aligned}$$

with the boundary conditions

$$\begin{aligned} \left\{ \begin{array} {l l l l l} {\varvec{{q}}} &  = 0, &  &  {\varvec{{x}}} \in \Gamma _0; \\ {\varvec{{q}}} &  = {\varvec{{q}}}_\Gamma , &  &  {\varvec{{x}}} \in \Gamma _{\textrm{n}}; \\ {\varvec{{q}}} \cdot {\varvec{{n}}} &  = h(u-u_{\textrm{env}}), &  &  {\varvec{{x}}} \in \Gamma _{\textrm{r}}; \end{array} \right. \end{aligned}$$

where the computational domain $\Omega$, which represents a cooling fin, is shown in Fig. 2, ${\varvec{{q}}}$ is the heat flux, *h* is the thermal conductivity, and ${\varvec{{q}}}_\Gamma = (q_\Gamma , 0)$ denotes the boundary heat source. Here, $\Gamma _{\textrm{n}}$ is the Neumann boundary, $\Gamma _0$ is the axis of symmetry and, hence, represents the adiabatic boundary, while the rest of the boundary, denoted by $\Gamma _{\textrm{r}}$, known as the Robin boundary, directly transfers heat to the environment and follows Newton’s cooling law. The conductivity is modeled as $\kappa (u, \varvec{\mu }) = \bar{\kappa } \exp {(-(\frac{u-\mu _0}{\mu _0})^2)}$, where $\mu _0 = 10$ is fixed. The parameter $\varvec{\mu } = [q_\Gamma , h, u_\textrm{env}, \bar{\kappa }] \in \mathbb {P}$, where the parameter domain $\mathbb {P} = [10,50] \times [1, 5] \times [0, 10] \times [5, 15] \subset \mathbb {R}^4$.

The weak formulation of (20) is then: Find $u \in H^1(\Omega )$ such that

22 $$\begin{aligned} \int _\Omega {{\varvec{q}}} \cdot \nabla v \, \textrm{d}V - h \int _{\Gamma _{\textrm{r}}} u v \, \textrm{d}S = - h u_{\textrm{env}} \int _{\Gamma _{\textrm{r}}} v \, \textrm{d}S - q_\Gamma \int _{\Gamma _{\textrm{n}}} v \, \textrm{d}S, \quad \forall v \in H^1(\Omega ). \end{aligned}$$

To test the efficiency of $\textrm{NN}_p$ as a hyper-reduction method, we apply the RBM, together with hyper-reduction, as discussed in Sections “Reduced basis method” - General hyper-reduction. The details of the related RB formulation are provided in Appendix B.

**Table 1:** Network architecture

**Network**	**Input**	**Intermediate**			**Output**
	$({\varvec{N}}^{{\varvec{r}}}{\varvec{+1}})$ **StdLy**	$({\varvec{N}}^{{\varvec{r}}}{\varvec{+1)}} {\varvec{\rightarrow }} {\varvec{80}}$ **StdLy**	${\varvec{80 \rightarrow 60}}$ **StdLy**	**60 ResNet**	${\varvec{Q}}^{{\varvec{r}}}$ **StdLy**
$\textrm{NN}_p$	1	1	1	3	1

“StdLy” means standard layer shown in Fig. 1b; “ResNet” means ResNet block shown in Fig. 1c; The number in the last row indicates the number of layers/blocks that sequentially constitute the network $\textrm{NN}_p$

*Numerical setting:* The (full-order) state, *u*, and derived quantity, ${\varvec{{q}}}$, have dimension $N^\textrm{f} = 1323$ and $Q^{\textrm{f}} = 4500$, respectively. The employed architecture of the second tier network, $\textrm{NN}_p$, is provided in Table 1. The network has more than 30 000 training parameters in total. Numerical tests show that this architecture of the neural network seems to achieve a good balance between size of the network and accuracy. If we increase the size of the neural network, e.g., have a wider or deeper network, the accuracy improves a little, but at the expense of a large computational cost. Increasing the capacity (i.e., the size of the NN) makes the optimization difficult and, thus, the generalization error may also increase since overfitting follows with the high network capacity; see the discussion in Chapter 5 of [28].

We also test another activation function design to replace the $\textrm{ELU}$ used in 2 standard layers for the proposed method by observing the function in (21). This modified activation function is defined as :

$$\begin{aligned} f(x) = x \exp {(-(x-1)^2)}. \end{aligned}$$

It retains a property of the $\textrm{ELU}$ and $\textrm{ReLU}$ in the sense that it outputs zero across a large part of its domain and, hence, it can also gain a sparse network as $\textrm{ReLU}$ and $\textrm{ELU}$ do. A drawback of this activation function is that it is not monotone and, hence, may cause some difficulties during the training stage [28]. We expect it to catch the exponentiality of the true model. According to our tests, in the context of this problem, the use of the modified activation function can slightly improve the accuracy of the prediction. However, the improvement is not so obvious: it only reduces the relative error by a factor of around three.

**Fig. 3:**
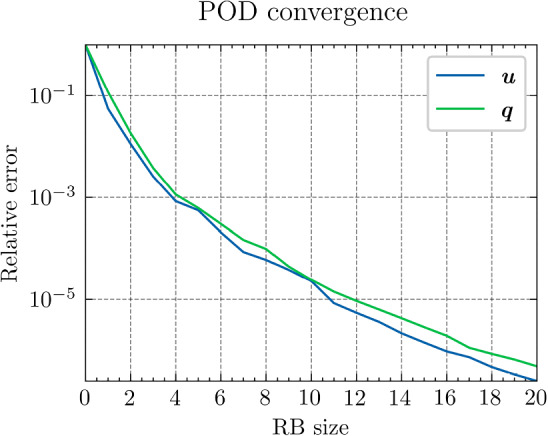
The convergence of the relative error of ${\varvec{{u}}}$ and ${\varvec{{q}}}$ w.r.t.  the reduced basis size ${N^{\textrm{r}}}$ and ${Q^{\textrm{r}}}$ by POD algorithm

*Results and discussion:* Using the POD algorithm to approximate the manifold of the unknown quantity ${\varvec{{u}}}$ and of the derived quantity ${\varvec{{q}}}$, we obtain the convergence curves shown in Fig. 3. We can empirically see that, for this problem, the Kolmogorov *N*-width^3^ [5] of both the manifolds of ${\varvec{{u}}}$ and ${\varvec{{q}}}$ are small, and the reduced-order models have high efficiency, which allows an efficient RBM (or TTDN). Furthermore, the convergence rates of the two quantities are roughly the same, indicating that $N^r$ is of the same order of magnitude as $Q^r$.

**Fig. 4:**
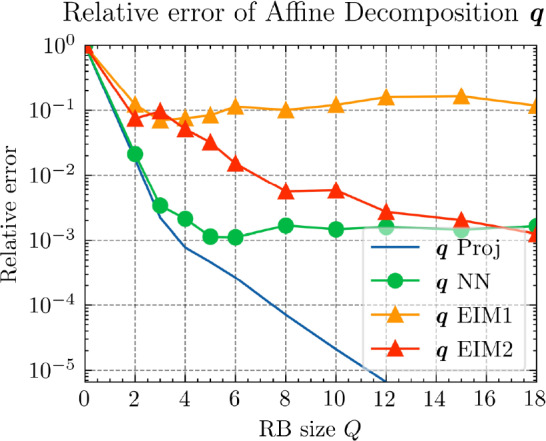
Comparison of the relative errors of ${\varvec{{q}}}$ of affine decomposition via $\textrm{NN}_p$ and via EIM

Figure 4 shows the relative approximation error of ${\varvec{{q}}}$, governed by (Baa), upon using the proposed second tier network $\textrm{NN}_p$ and EIM (under different training data). The blue curve “${\varvec{q}}$” Proj error of projection into the reduced space $\{\xi _p^{\textrm{RB}}\}_{p=1}^{Q^{\textrm{r}}}$ and serves as a reference. As a comparison, the curves EIM1 and EIM2 are the relative errors obtained by EIM under two different sizes of training data. EIM1 is based solely on the POD training data, with $M=1500$ and a required memory or storage space of 80MB. EIM2, on the other hand, utilizes a larger dataset with *M* on the order of $\mathcal {O}(10^4)$, necessitating significantly more storage space—850MB. Consequently, the offline training time for EIM2 is approximately an hour and a half, substantially longer than that for EIM1, as detailed in Table 2. For $\textrm{NN}_p$, the smaller storage requirement per training point permits the generation of a substantially larger training dataset, with size $M \sim \mathcal {O}(10^5)$ and a storage space of 41MB. The additional time required to generate this data is 400s (This is not included in the table). Even when accounting for this auxiliary time, $\textrm{NN}_p$ exhibits a much shorter overall training duration than EIM2. Regarding prediction time, it remains constant irrespective of the training data size, thus EIM1 and EIM2 share identical prediction times. Both are significantly slower than $\textrm{NN}_p$, by a factor of $\mathcal {O}(10^2)$.

**Table 2:** Training data and training time

**Method**	**Storage space**	**Training time**	**Predict time**
$\textrm{NN}_p$	41MB	< 300s	< 2ms
EIM1	80MB	$\sim$ 600s	$\sim$ 150ms
EIM2	850MB	$\sim$ 5400s	$\sim$ 150ms

**Fig. 5:**
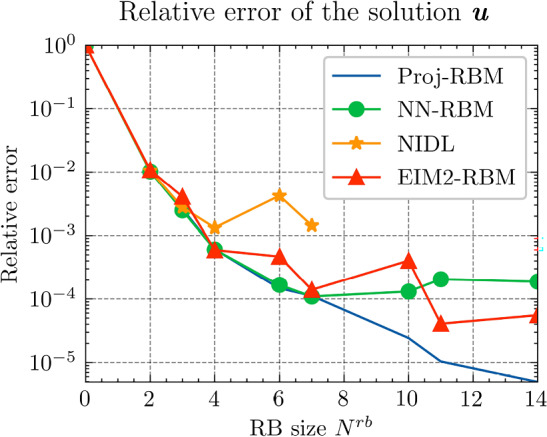
The relative errors of the final results of ${\varvec{{u}}}$ by different methods

In Fig. 4, we observe that hyper-reduction via $\textrm{NN}_p$ achieves high efficiency. With fewer than 6 terms, it attains an accuracy of $10^{-3}$, surpassing EIM2, which requires 12 terms to reach a similar level of accuracy. Nonetheless, due to the inherent regression error limitations of the neural network, the relative error plateaus at approximately ${1.5\times 10^{-3}}$ with 6 terms, showing no further decrease. In contrast, EIM2 attains a slightly lower relative error of ${1.2\times 10^{-3}}$ with 18 RB terms. Furthermore, EIM1, while having an offline cost comparable to $\textrm{NN}_p$, suffers from limited generalization capacity, resulting in an accuracy of only ${10^{-1}}$.

Next, we conduct a performance comparison between the RBM enhanced by the proposed hyper-reduction method $\textrm{NN}_p$, denoted as NN-RBM, and EIM with the EIM2 setting, denoted as EIM2-RBM. Additionally, we include a non-intrusive deep learning method (NIDL) proposed in [56] in our comparative analysis. NIDL is a deep learning based MOR method which trains a deep network to learn and predict the residual of the PDE, and then applies Newton’s method to determine the solution of the PDE. For more details, please refer to [56]. For NIDL, we adopt a network structure similar to that described in [56], ensuring the computational costs of NN-RBM and NIDL are closely matched. The findings, presented in Fig. 5, elucidate the respective performance metrics. The main observations are as follows.

(i) Initially, all three methods exhibit similar relative error decay rates, with the accuracy predominantly influenced by the projection error as indicated by the Proj-RBM curve.
(ii) The curves for Proj-RBM and NN-RBM overlap for $N^{\textrm{r}} \le 7$, suggesting that the error attributable to the neural network in NN-RBM is negligible in comparison to the projection error.
(iii) NN-RBM outperforms EIM2-RBM until $N^{\textrm{r}} = 11$, beyond which EIM2-RBM exhibits superior performance.
(iv) NIDL encounters divergence issues with Newton’s method for $N^{\textrm{r}} > 7$ for this specific problem^4^. However, both the proposed method and EIM maintain good convergence.

Referring back to the computational costs detailed in Table 2, it is observed that the neural network is 75 times faster than EIM during the online phase, thus positioning the proposed NN-RBM as the most efficient method at the error level of ${10^{-4}}$ or larger.

In summary, we observe that, as a general hyper-reduction technique, the proposed method outperforms EIM in the sense of higher accuracy and less space and time complexity in the training and prediction stages for small RB sizes. In particular, for very small RB sizes, the resulting accuracy for both ${\varvec{{q}}}$ and *u* by the proposed method is very close to the projection accuracy.

#### Non-affine heat transfer problem

**Fig. 6:**
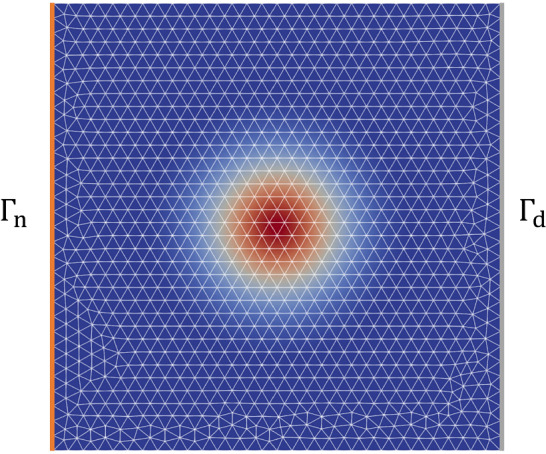
The domain $\Omega$, the mesh and $\kappa$ at $\varvec{\mu } = (0,0)$, for the non-affine problem in Subsection Experiments for TTDN

**Problem description:** In this section, we consider a linear but non-affine problem—a heat transfer problem where the medium has a parameterized spatial distribution. This problem is mathematically described by the following governing equations:

$$\begin{aligned}&- \nabla \cdot {\varvec{{q}}}({\varvec{{x}}}) = 0, \quad {\varvec{{x}}} \in \Omega ; \\&{\varvec{{q}}} = - \kappa ({\varvec{{x}}}; \varvec{\mu }) \nabla u({\varvec{{x}}}), \quad {\varvec{{x}}} \in \Omega ; \end{aligned}$$

with the boundary conditions

$$\begin{aligned} \left\{ \begin{array} {l l l l} {\varvec{{u}}} &  = 0, &  &  {\varvec{{x}}} \in \Gamma _d; \\ {\varvec{{q}}} \cdot {\varvec{{n}}} &  = 0, &  &  {\varvec{{x}}} \in \Gamma _0; \\ {\varvec{{q}}} \cdot {\varvec{{n}}} &  = q_\Gamma , &  &  {\varvec{{x}}} \in \Gamma _{\textrm{n}}, \end{array} \right. \end{aligned}$$

where the computational domain is a unit square $\Omega = (-0.5, 0.5) \times (-0.5, 0.5)$ with the Dirichlet boundary $\Gamma _d$, (non-zero) Neumann boundary $\Gamma _{\textrm{n}}$ shown in Fig. 6. The (fixed, zero) Neumann $\Gamma _0$ represents the rest of the domain boundary. The heat transfer medium has a conductivity distribution that is given by

$$\begin{aligned} \kappa ({\varvec{{x}}}; \varvec{\mu }) = \kappa _0 + \bar{\kappa } \exp {\left( -\frac{({\varvec{{x}}}-\varvec{\mu })^T \cdot ({\varvec{{x}}}-\varvec{\mu })}{\mu _0^2}\right) }, \end{aligned}$$

which simulates a high conductivity with a fixed size of $\mu _0$ appearing at the location $\varvec{\mu }$ and attenuating as the distance increases. The parameter $\varvec{\mu } = [\mu _1, \mu _2] \in \mathbb {P} = [-0.45,-0.15] \times [-0.15, 0.15]$. Fig. 6 shows an example of $\kappa$ when $\varvec{\mu } = (0, 0)$. Here, we fix $q_\Gamma = 25$, and $\kappa _0 = 5$, $\bar{\kappa } = 10$, $\mu _0 = 0.15$.

As indicated in Subsect. “General hyper-reduction”, the affine matrix formulation of this problem can be directly written as:

$$\begin{aligned} \left( \sum _{p=1}^{Q^{\textrm{r}}} \theta _p(\varvec{\mu }) {\textbf {K}}_p \right) \cdot {\textbf {u}} = {\varvec{{f}}}, \end{aligned}$$

where ${\textbf {K}}_p \in \mathbb {R}^{N^{\textrm{r}} \times N^{\textrm{r}}}$ and ${\varvec{{f}}} \in \mathbb {R}^{N^{\textrm{r}}}$ such that, for $p \in (1, ..., Q^{\textrm{r}})$,

$$\begin{aligned} {[}  {\textbf {K}}_p]_{j,i} = \int _{\Omega } \xi _p^{\textrm{RB}} \nabla \phi _i^{\textrm{RB}} \cdot \nabla \phi _j^{\textrm{RB}} \,\textrm{d}V, \qquad ({\varvec{{f}}})_j = \int _{\Gamma _{\textrm{n}}} q_\Gamma \phi _j^{\textrm{RB}} \,\textrm{d}S. \end{aligned}$$

We thus need the map $\varvec{\theta }_q(\varvec{\mu })$ that achieves the approximated affine decomposition via  or via, e.g., the EIM.

*Numerical setting:* The full-order $Q^{\textrm{f}}$ and $N^{\textrm{f}}$ corresponding to the mesh in Fig. 6 are $N^{\textrm{f}} = Q^{\textrm{f}} = 1126$. In this problem, the number of input features is only 2 and the parameter range $\mathbb {M} = [-0.45,-0.15] \times [-0.15, 0.15]$ is also small. Hence, the required size of the training data, i.e., *M*, is small compared to the previous numerical experiment. We use $M = 1500$ parameter samples for the POD algorithm. The architecture of network $\textrm{NN}_p$ is given in Table 3. The number of training parameters for the network is dependent on $Q^\textrm{r}$ and is around 10000 for all $Q^\textrm{r}$.

**Table 3:** Network architecture

**Network**	**Input**	**Intermediate**			**Output**
	**2** **StdLy**	$\varvec{2 \rightarrow 10}$ **StdLy**	$\varvec{10\rightarrow 25}$ **StdLy**	$\varvec{25}$ **ResNet**	$\varvec{Q}^{\textbf{r}}$ **StdLy**
$\textrm{NN}_p$	1	1	1	7	1

**Fig. 7:**
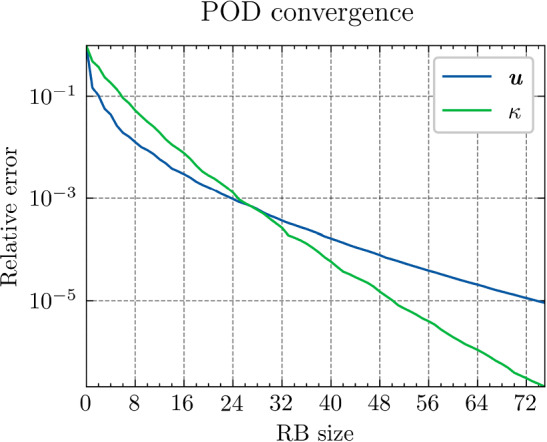
The convergence of the relative error of ${\varvec{{u}}}$ and $\kappa$ with respect to the reduced basis size ${N^{\textrm{r}}}$ and ${Q^\textrm{r}}$ by POD algorithm

**Results and discussion:** The decay curves obtained by applying the POD algorithm to ${\varvec{{u}}}$ and to $\kappa$ are shown in Fig. 7. For this problem, this error decays relatively slowly, which means that the Kolmogorov *N*-width is larger compared to the previous example. This is the case since we are trying to linearly approximate the Gaussian functions with different center points, i.e., different locations of peak. Such a problem is known to pose a challenge for standard MOR techniques.

Since the training data is small, the offline cost for training the $\textrm{NN}_p$ and the EIM are similar, and both of them take less than 200s. Furthermore, the memory complexity for training $\textrm{NN}_p$ is two orders of magnitude smaller than EIM. For the EIM, the prediction time for 320 test points is around 0.5s, while, for the proposed approach, the prediction takes around 2.3ms. The approach by $\textrm{NN}_p$ is again faster than the EIM; see Table 4.

**Table 4:** Training data and training time

**Method**	**Training time**	**Prediction time**
${{NN}}_{{p}}$	${{<}}$ 200 s	${{<}}$ 2.3 ms
EIM	${{<}}$ 200s	${\sim }$ 500ms

**Fig. 8:**
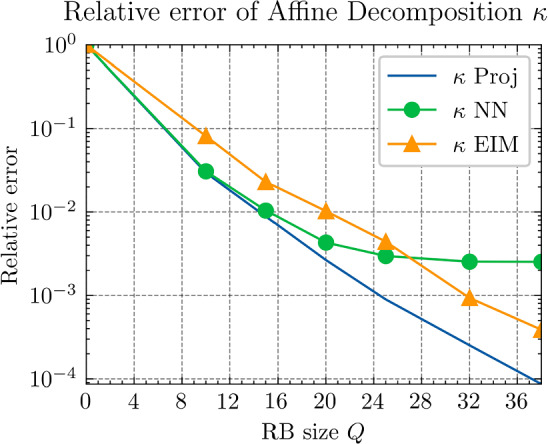
The relative errors of $\kappa$ of affine decomposition by $\textrm{NN}_p$ and by the EIM

Figure 8 shows the comparison of the relative error of $\kappa$ obtained using the EIM and the proposed $\textrm{NN}_p$. The blue curve $\kappa$ Proj shows the projection error which represents the best achievable error. The observed behaviour is similar to that in Fig. 4 for the previous example. However, due to the difficulty of the linear approximation of Gaussians, the approximated affine decomposition by $\textrm{NN}_p$ and the EIM are not efficient—more than 15 terms are needed to achieve an accuracy of ${10^{-2}}$. Compared to the EIM, the NN-based approach does not show a large improvement. It can economise around 5 terms for the same accuracy. However, the error stagnates with the increasing number of terms when it reaches $\sim$ $2.1\times {-3}$, an accuracy limitation of neural network. When the number of terms is less than 27, the proposed method is more efficient than the EIM. The other advantage is that, as mentioned above, the prediction time using the proposed method is 200 times faster than the EIM.

**Fig. 9:**
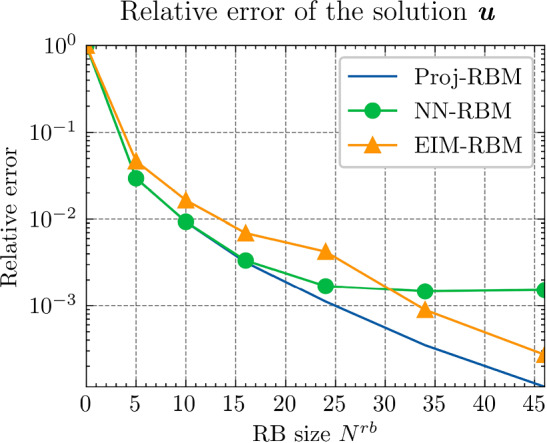
The relative errors of the final results of ${\varvec{{u}}}$ by the proposed $\textrm{NN}_p$ and by the EIM

Figure 9 shows the relative error of the solution ${\varvec{{u}}}$ by leveraging the MOR and the approximated affine decomposition via $\textrm{NN}_p$ and EIM, namely NN-RBM and EIM-RBM, respectively. The Proj-RBM is also the accessible lower bound error. The curves show the same behavior as that in Fig. 8. Hence, the discussion above for $\kappa$ is also applicable for ${\varvec{{u}}}$.

In summary, we observe that, the proposed method outperforms EIM again as an approximated affine decomposition method, in the sense of higher accuracy and less space and time complexity when the reduced basis size is relatively small. However, when the reduced basis size is large, EIM will surpass the proposed method since the achieved accuracy of the later is limited by the accuracy of the neural network.

#### Time dependent Burgers’ problem

*Problem description:* In this experiment, we consider a time-dependent, one-dimensional, viscous Burgers’ equation [56] that is governed by:

$$\begin{aligned} \begin{aligned} \frac{\partial u({\varvec{{x}}}, t)}{\partial t} + u({\varvec{{x}}}, t) \nabla u({\varvec{{x}}}, t) -&\mu _1 \nabla \cdot \nabla u({\varvec{{x}}}, t) - \mu _2 \exp {(\mu _3 {\varvec{{x}}})} \\&= 0, \quad {\varvec{{x}}} \in \Omega = [0,1], \ t \in [0, T=1], \end{aligned} \end{aligned}$$

together with the initial and boundary conditions

$$\begin{aligned}&u({\varvec{{x}}}, t = 0) = 0; \\&u({\varvec{{x}}} = 0, t) = u({\varvec{{x}}} = 1, t) = 0. \end{aligned}$$

**Fig. 10:**
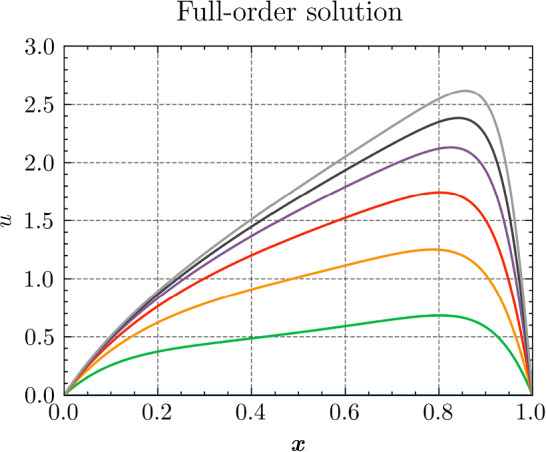
The full-order solution at $\mu$= (0.1, 3, 1), at *t* = 0 s, 0.11 s, 0.22 s, 0.33 s, 0.44 s, 0.55 s and 1 s, from bottom to top

The model problem, discretization scheme, and other settings are the same as employed in [56]: $\varvec{\mu } = (\mu _1, \mu _2, \mu _3) \in \mathbb {P} = [0.01, 0.1] \times [2, 3] \times [0, 1]$. The computational domain $\Omega$ is discretized by 200 finite elements and, hence, $N^{\textrm{f}} = 201$. For the temporal discretization, we use the two-stage, second order diagonally implicit Runge-Kutta method (DIRK) [79], and discretize [0, *T*] into $N^T = 100$ uniform time steps, with the step length denoted by $\delta t$. An example of the solution at $\varvec{\mu } = (0.1, 3, 1)$ is shown in Fig. 10.

**Fig. 11:**
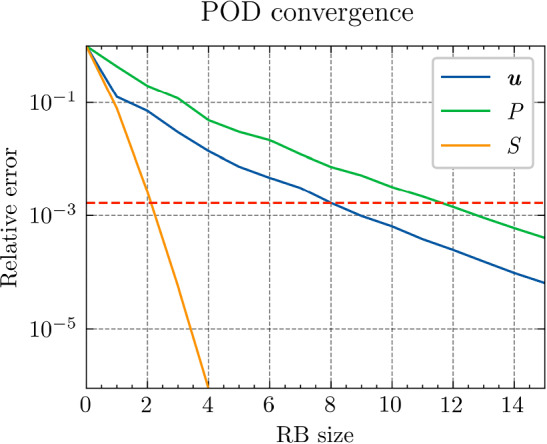
The decay of the relative error in the POD approximation of *u*, *P* and *S* with respect to ${N^{\textrm{r}}}$, ${Q^{\textrm{r}}_1}$ and ${Q^\textrm{r}_2}$. The red dashed line shows the accuracy of ${\varvec{{u}}}$ at $N^{\textrm{r}} = 8$

In this problem, we need to apply general hyper-reduction twice— one for the non-linearity $P = u \nabla u$, and another for the non-affine term of $S = \exp {(\mu _3 {\varvec{{x}}})}$. We consider that the former is approximated by the reduced basis space $\{\xi _p^{\textrm{RB}}\}_{p=1}^{Q^{\textrm{r}}_1}$ and the coefficient vector $\varvec{\theta }({\textbf {u}}^{\textrm{RB}})$, and that the latter is approximated by the reduced basis space $\{\zeta _p^\textrm{RB}\}_{p=1}^{Q^{\textrm{r}}_2}$ and the coefficient vector $\varvec{\beta }(\mu _3)$. Both of the coefficient vectors are approximated by their individual networks,  and . In addition, the reduced basis space for *u* is $\{\phi _i^{\textrm{RB}}\}_{i=1}^{N^{\textrm{r}}}$. The details of the RB formulation with DIRK is presented in Appendix C.

*Numerical setting:* Here, we set $N^{\textrm{f}} = Q^{\textrm{f}}_2 = 201$, $Q^{\textrm{f}}_1 = 200$. The decay curves obtained by applying the POD algorithm to *u*, *P*, and *S* are shown in Fig. 11. Similar to [56], we set $N^{\textrm{r}} = 8$ for *u*. To achieve the same accuracy ($\sim$ ${10^{-3}}$) of *P*, we choose $Q^{\textrm{r}}_1 = 12$ (see the red horizontal dashed line in Fig. 11); for *S*, we set $Q^{\textrm{r}}_2 = 4$. For the training data, we use a $3 \times 3 \times 3$ uniform sample for $\mathbb {P}$, and obtain 2700 full-order snapshots as the training data for the POD algorithm, $\textrm{NN}_p^1$, and $\textrm{NN}_p^2$. The test sample is a $5 \times 5 \times 5$ uniform sample in $\mathbb {P}$ minus the points that exist in the training data. Thus, there are 98 test points (or 9800 if we count the time steps).

**Table 5:** First network architecture (DC)

**Layer width**									
NILD	11	80	120	240	480	240	120	80	8
$\textrm{NN}_p^1$	8	40	80	120	240	120	80	40	12
$\textrm{NN}_p^2$	1	10	20	30	60	30	20	10	4

**Table 6:** Second network architecture (ResNet)

**Network**	**Input**	**Intermediate**		**Output**
NILD	11 StdLy	$11 \rightarrow 60$ StdLy	60 ResNet	8 StdLy
	1	1	3	1
$\textrm{NN}_p^1$	8 StdLy	$8 \rightarrow 40$ StdLy	40 ResNet	12 StdLy
	1	1	3	1
$\textrm{NN}_p^2$	1 StdLy	$1 \rightarrow 10$ StdLy	10 ResNet	4 StdLy
	1	1	3	1

**Table 7:** Numbers of the training parameters of the network

**Method**	**DC-NIDL**	**DC-NNRBM**	**Res-NIDL**	**Res-NNRBM**
Number of parameters	310088	90166	23168	11416

Two different network architectures are used in this example. In line with the structure described in [56] for the NIDL method, we employ a diverging-converging (DC) network as the first architecture. It involves an up-scaling and a down-scaling sub-network and does not contain ResNet. The corresponding architectures are shown in Table 5. We use ResNet as the second architecture; see Table 6. The number of the training parameters for the methods under consideration are shown in Table 7, where the DC network with the NIDL method and with the proposed NN-RBM is denoted by DC-NIDL and DC-NNRBM, respectively. The ResNet with the NIDL method and with the NN-RBM is denoted by Res-NIDL and Res-NNRBM, respectively. Similar to the experiment in [56], we also implement the EIM for this problem with the same training data. This method, in the sequel, is denoted by the EIM-RBM. Note that, in the experiments, all the networks have the same depth if we regard a ResNet layer as 2 standard layers. The second architecture with ResNet has less number of parameters due to the small width of the network.

**Fig. 12:**
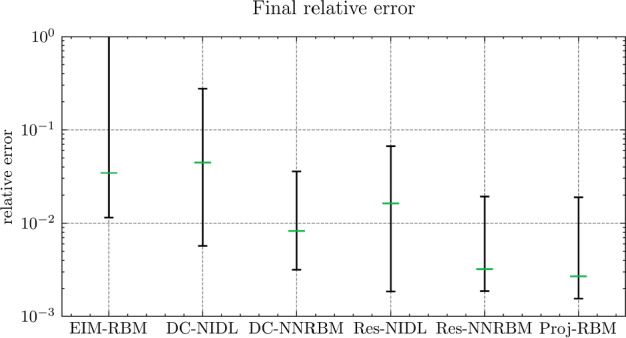
Comparison of the final relative error for the test set for each numerical method. The horizontal bars from top to bottom on each vertical line represent the max, mean and min relative error, respectively. Res-NNRBM is the proposed method; DC and Res refer to different architectures of the neural network. EIM is unstable during the temporal succession hence the max error is larger than 1. According to Table 7, the sizes of networks decrease but the last, cheapest one, corresponding to the proposed Res-NNRBM, has the lower error, which is close to the best achievable error resulting from the expensive Proj-RBM

*Results and discussion:* The minimum, maximum, and median relative errors for the test set are shown in Fig. 12. In our numerical tests, the four NN-based methods have neither suffered from instability during the time step succession, nor suffered from the divergence issues during the Newton iterations of (C5). Note that, in Fig. 12, according to Table 7, the number of parameters of the network of the four methods decreases from the left to the right, which indicates that the computational complexity is also decreasing.

Figure 12 shows that the NN-RBM provides nearly 5 times improvement to the accuracy compared to that resulting from the NIDL method, with less than half the number of the training parameters. Also, the application of ResNet can contribute around a factor of three improvement to the accuracy over the DC network, with less than one-eighth of the training parameters. The proposed Res-NNRBM, with $\textrm{NN}_p$, compared to the DC-NIDL method, achieves the improvement of more than 10 times to the accuracy of the median. Furthermore, the total number of training parameters, i.e., the size of the proposed network, are only $\frac{1}{27}$ of the size of the network used in DC-NIDL.

The instability for the EIM-RBM during the time succession, which appears in [56], is also reproduced, but the median error of the EIM-RBM does not suffer from this instability and is only comparable to the median error via DC-NIDL method. Compared to Res-NNRBM, EIM-RBM performs even worse. Hence, we reach the same conclusions, as in [56], that the EIM-RBM does not work well for the dynamical system at hand.

The last bar in Fig. 12 shows the relative error of the projection method as the best error that one can seek to achieve. From Fig. 11, we recall that, for $N^{\textrm{r}} = 8$, the relative projection error of the POD algorithm is ${1.67\times ^{-3}}$, another lower bound limited by reduced basis space setting. The proposed Res-NNRBM can get the approximate solutions with accuracy very close to the aforementioned lower bound. Hence, we can conclude that the second tier of the TTDN performs well as an affine decomposition or hyper-reduction method in this example.

In summary, the proposed method, which serves as a hyper-reduction technique, achieves better accuracy than the NIDL method for all the network architecture configurations, even with fewer network parameters compared to that in the NIDL.

### Experiments for TTDN

In this section, we perform numerical experiments to show the efficiency of the proposed TTDN method compared to the PDNN method, i.e., the network proposed in [40].

#### Non-linear heat transfer problem

In this numerical experiment, we reuse the non-linear heat transfer model problem discussed in Sect. “Non-linear heat transfer problem”. Here, the unknown quantity of interest *u* is the temperature field, and the derived quantity *P* (referred to as ${\varvec{{q}}}$ in Subsect. “Non-linear heat transfer problem”) is the heat flux. We directly use the network $\textrm{NN}_p$, which we trained as a hyper-reduction tool in Sect. “Non-linear heat transfer problem”, to show the different performance of the two possible ways of perceiving the TTDN method; see Subsect. “Connection to existing methods”.

*Numerical setting:* The dimensionality of parameter $\varvec{\mu }$ is $\dim (\varvec{\mu })=4$. The reduced bases are obtained by the POD algorithm. The reduced dimension of *u* and of *P* are, respectively, set to $N^{\textrm{r}} = Q^{\textrm{r}} = 6$, which, according to Fig. 3, corresponds to the projection error of less than $5\times ^{-4}$. This error acts as the best achievable error for this experiment. The computational cost of a full-order solution, i.e., a training point for the PDNN, in this experiment is around 250 times the computational cost of the evaluation of $(\varvec{\mu }, {\textbf {u}}^{\textrm{RB}}) \mapsto {\textbf {P}}^{\textrm{RB}}$, i.e., a training point for $\textrm{NN}_p$. It thus satisfies the assumption that the evaluation $(\varvec{\mu }, {\textbf {u}}^{\textrm{RB}}) \mapsto {\textbf {P}}^\textrm{RB}$ is much cheaper than computing the full-order solution. The time required for the generation of the full-order data is around 1200s for 1500 full-order solutions, while the time needed for generating the $(\varvec{\mu }, {\textbf {u}}^{\textrm{RB}}) \mapsto {\textbf {P}}^\textrm{RB}$ data is around 100s. This means that the size of the latter (the constitutive law data) is around 21 times the size of the former (full-order data). However, the computational cost for the large constitutive law data is still much less than the cost for the small full-order data.

The architectures of the networks are presented in Table 8. Note that, the second tier of TTDN, i.e., $\textrm{NN}_p$, is set to be the $\textrm{NN}_p$ that was used as the hyper-reduction tool in Subsect. “Non-linear heat transfer problem”.

**Table 8:** Network architecture

**Network**	**Input**	**Intermediate**		**Output**
${{{NN}}}_{{u}}^{{{pdnn}}}$	4 StdLy	$4 \rightarrow 50$ StdLy	50 ResNet	6 StdLy
	1	1	5	1
$\textrm{NN}_u$	4 StdLy	$4 \rightarrow 50$ StdLy	50 ResNet	6 StdLy
	1	1	5	1
$\textrm{NN}_p^{\textrm{pdnn}}$	4 StdLy	$4 \rightarrow 50$ StdLy	50 ResNet	6 StdLy
	1	1	5	1
$\textrm{NN}_p$	5 StdLy	$5 \rightarrow 80 \rightarrow 60$ StdLy	60 ResNet	6 StdLy
	1	1	3	1

In the sequel, TTDN1 means that the results are obtained by the TTDN method using the first option, while TTDN2 denotes the same using the second one. To be more precise, in TTDN1, after the pretraining of $\textrm{NN}_u$ and $\textrm{NN}_p$, we combine them together to build $\textrm{NN}_t$ and then use the semi-supervised learning strategy to train the whole $\textrm{NN}_t$, which means that all the training parameters of $\textrm{NN}_t$, including the parameters of $\textrm{NN}_u$ and $\textrm{NN}_p$, are trained. In TTDN2, $\textrm{NN}_p$, which is trained as the hyper-reduction tool in Subsect. “Non-linear heat transfer problem”, is believed to approximate the constitutive law $P(u; \varvec{\mu })$ with high quality. Hence, it is only applied as a part of the loss function in (19). Thus, its training parameters are not trained while training $\textrm{NN}_t$, which means that we train $\textrm{NN}_t$ in a semi-supervised manner. In other words, only the training parameters of $\textrm{NN}_u$ are trained, while all the parameters of $\textrm{NN}_p$ are frozen. This is corresponding to the second training option for TTDN, see Subsect. “Connection to existing methods” for more details.

**Fig. 13:**
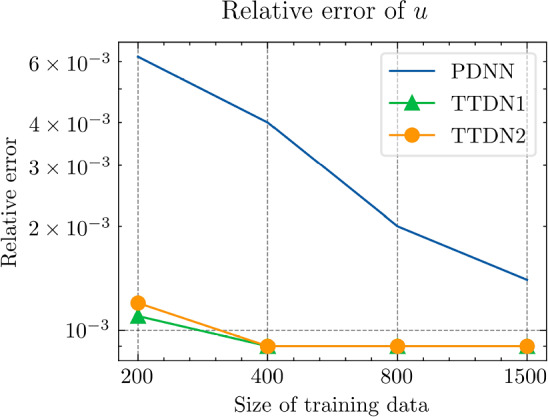
The generalization relative error of *u* for different methods and size of training data

**Fig. 14:**
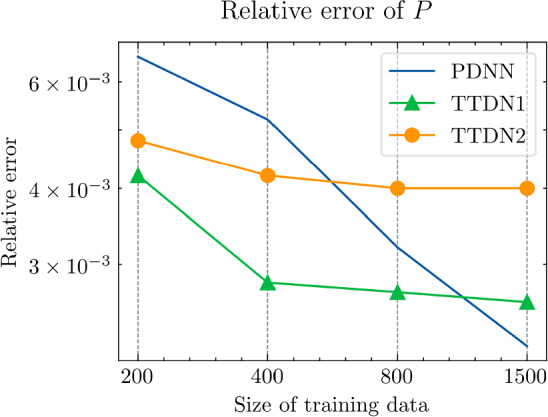
The generalization relative error of *P* for different methods and size of training data

**Fig. 15:**
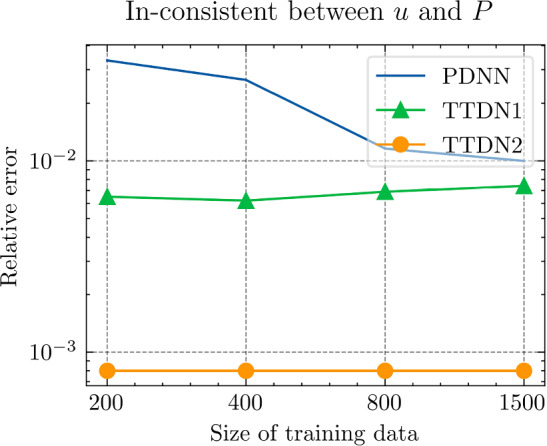
The inconsistency between *u* and *P* for different methods and size of training data

*Results and discussion:* To show the efficiency of the (two variants of the) TTDN method over the PDNN method, we draw the comparison of their performance with different sizes of the full-order training data. The different training options of the TTDN method lead to different means to train and, subsequently, different results in, e.g., the accuracy of *P* and the consistency between *u* and *P*. The results are shown in Figs. 13, 14, and 15. In these figures, the curves show the results obtained using different methods. The x-axis in Figs. 13, 14, and 15 shows the size of different training data $M_U$ used to train the PDNN and to pretrain the $\textrm{NN}_u$ of the TTDN. From Fig. 13, we can see that, for the quantity of interest *u*, the TTDN method always has much higher accuracy compared to the PDNN method. This holds even for the smaller size, $M_U$, of the full-order data. The accuracy of the TTDN method with the smallest size of the full-order data is better than the PDNN method with the largest size. However, the increase of $M_U$ does not improve the accuracy of the regression of the TTDN method. According to Chapter 5 of [28], in this case, the gap of the generalization error and the training error is small, and the capacity of the network limits the accuracy.

For the derived quantity *P*, in Fig. 14, we see that, when the training data is limited, the proposed TTDN method has a better performance than the PDNN method. However, when the training data is large enough ($M_U = 1500$ is already large enough in the scope of this experiment), the PDNN method will defeat the TTDN one. When the training data is large enough that it contains almost all the information in the parameter space, the generalization error of the PDNN method can be small. In Fig. 14, we also find that TTDN1 works better than TTDN2. This can be explained by an error propagation analysis as discussed next.

Assume that the error of *u* is $\delta u$, and the constitutive law is given by $\mathcal {C}: (u; \varvec{\mu }) \mapsto P$. We then have $\Vert P^{\textrm{true}}\Vert = \Vert \mathcal {C}(u^\textrm{true}; \varvec{\mu })\Vert = \Vert \mathcal {C}(u+\delta u; \varvec{\mu })\Vert \approx \Vert \mathcal {C}(u; \mathcal {\mu })\Vert + c \Vert \delta u\Vert$, where we assume that $\mathcal {C}$ is Lipschitz continuous and *c* is bounded by the Lipschitz constant of $\mathcal {C}$. In our numerical experiment, introducing a fluctuation $\delta u \approx {10^{-3}}$ to the true constitutive map $\mathcal {C}$ leads to the fluctuation in *P* in the level of ${4\times 10^{-3}}$. This means that $c \approx 4$. The resulting relative error in *P* is very close to the error in the TTDN2 curve in Fig. 14, showing that $\textrm{NN}_p$ approximates well the true map $\mathcal {C}$. Via the second training option, since $\textrm{NN}_p$ is frozen, reducing the error in *P* will require simultaneously reducing the error in *u*. Given that further improving the accuracy in *u*, whose relative error is now ${e-3}$, is difficult, it is challenging to improve the accuracy of *P*. Conversely, using the first training option, it is possible for the optimizer to adjust the trainable parameters in $\textrm{NN}_p$ to improve the accuracy of *P*, without requiring the error in *u* to be reduced at the same time.

However, the adjustment of the trainable parameters of $\textrm{NN}_p$ will break the “good enough” $\textrm{NN}_p$ in the pretraining stage. Consequently, the perfect consistency between the resulting *u* and *P* due to the good approximation of $\textrm{NN}_p$ to $\mathcal {C}$ will also be broken. In this work, we define the inconsistency between *u* and *P* as:

23 $$\begin{aligned} \frac{\Vert P - \mathcal {C}(u; \varvec{\mu })\Vert }{\Vert \mathcal {C}(u; \varvec{\mu })\Vert }, \end{aligned}$$

where *P* and *u* denote the results predicted by each method. This quantity denotes how different the predicted *P* is to the one obtained by the true constitutive law and the predicted *u*. The lower it is, the better the results are in the sense that the obtained *u* and *P* follow the underlying physical laws. Fig. 15 shows the inconsistency between predictive *u* and *P* using PDNN and TTDN. We can see, that, as discussed above, the second training option for TTDN, with a good enough pretrained $\textrm{NN}_p$, has the best consistency between *u* and *P*. The training of $\textrm{NN}_t$ in accordance with the original version, shown in TTDN1, breaks the consistency but is still better than the PDNN. This option makes a moderate compromise on consistency to achieve better accuracy of *P*. The PDNN method has the worst consistency between *u* and *P* since it trains the networks $\textrm{NN}_u^{\textrm{pdnn}}$ and $\textrm{NN}_p^{\textrm{pdnn}}$ individually and does not consider the underlying relation between *u* and *P* at all.

In conclusion, in this experiment, TTDN1 performs better than PDNN, except for the case that the full-order data is large, where the accuracy of the derived quantity *P* is worse than that obtained using the PDNN method. However, for the unknown quantity of interest *u*, the proposed TTDN method performed much better than the PDNN method with at most 1500 training data points, even when the TTDN is trained with very limited full-order training data. In the numerical tests, the TTDN2 has the best consistency between *u* and *P*.

#### Hyper-elasticity problem in multi-scale framework

This example is based on a multi-scale setting in mechanics; for related details, please refer [80–83]. For the application of machine learning to multi-scale modeling, we refer the reader to the review work [84].

**Fig. 16:**
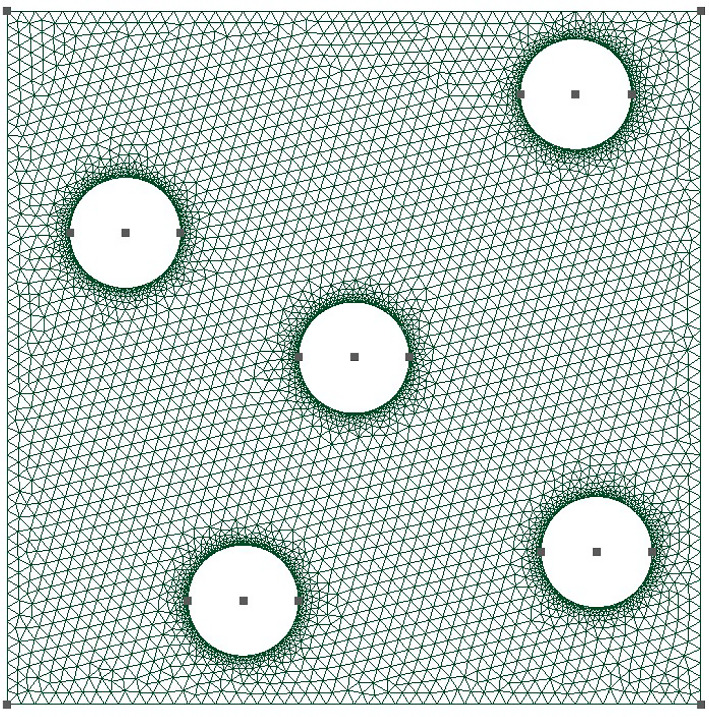
The computational domain and the mesh that describes the micro-scale porous model

*Problem description:* The computational homogenization method, a commonly employed numerical technique, uses the finite element squared method that separates solving the PDE (see (D7)) into the micro-scale simulation and the macro-scale simulation; see the details in [80–83]. In particular, for the mechanics problem, the micro-scale simulation aims at computing the effective macro-scale stress ${\textbf {P}}_{\textrm{M}}({\textbf {F}}_{\textrm{M}}; \lambda )$ and the effective stiffness tensor ${\textbf {K}}_{\textrm{eff}} ({\textbf {F}}_{\textrm{M}}; \lambda )$ within the representative volume element, denoted as $\Omega _{\textrm{m}}$ and presented in Fig. 16, for a parameter vector that is concatenated by the macro-scale strain ${\textbf {F}}_{\textrm{M}}$ and the Lamé parameter $\lambda$ (see the problem description below). Subsequently, the macro-scale simulation solves the whole problem by using the numerical effective constitutive law on the macroscopic computational domain $\Omega$. In this work, we are interested in the micro-scale problems characterized by non-linear output functionals, ${\textbf {P}}_\textrm{M}$ and ${\textbf {K}}_\textrm{eff}$, of unknown micro-scale displacement $u_\textrm{m}$. For the complete implementation of multi-scale simulation with TTDN [86].

**Fig. 17:**
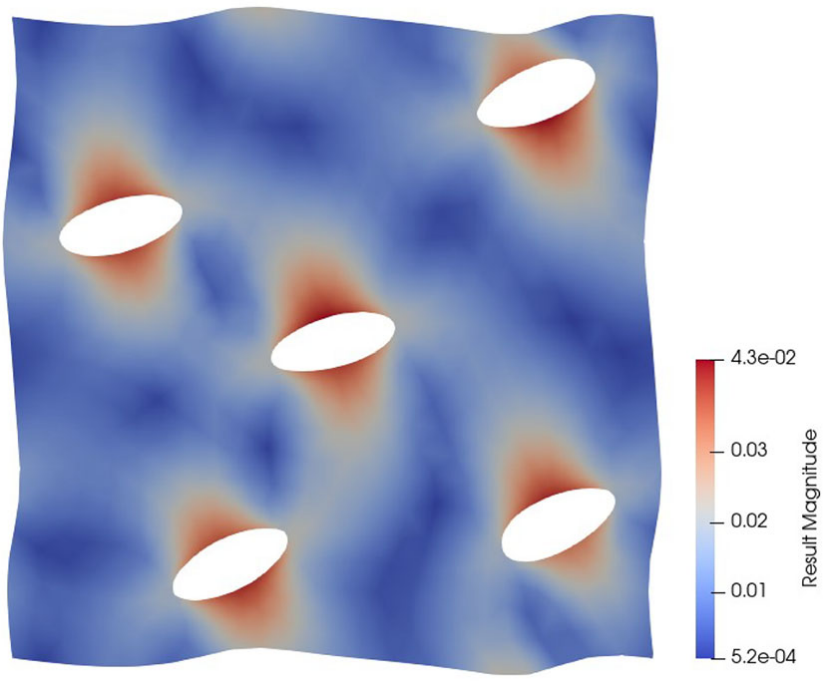
An example of the displacement $\tilde{{\varvec{{u}}}}$. The results are at a magnification of 3

The micro-scale PDE, implemented in the micro-scale $\Omega _{\textrm{m}}$ that represents a point in $\Omega$, reads: For a given macro-scale strain ${\textbf {F}}_{\textrm{M}}$ and Lamé parameter $\lambda$, find the micro-scale displacement ${\varvec{{u}}}_{\textrm{m}} \in H^1(\Omega _{\textrm{m}}) \times H^1(\Omega _{\textrm{m}})$, such that

24 $$\begin{aligned} \mathrm{div_m \,}{\textbf {P}}_{\textrm{m}} ({\varvec{{u}}}_{\textrm{m}}; \lambda ) = 0, \end{aligned}$$

complemented with the periodic boundary condition for ${\varvec{{u}}}_{\textrm{m}}$ and an anti-periodic boundary condition for the micro-scale stress ${\textbf {P}}_{\textrm{m}}$, holds. Note that these periodic boundaries depend on the micro-scale strain ${\textbf {F}}_{\textrm{M}}$. The details of the multi-scale hyper-elastic model are shown in Appendix D, and the illustration of the periodicity is presented in Fig. 17.

In this problem, the input parameter $({\textbf {F}}_{\textrm{M}}, \lambda )$ has dimension 4 since ${\textbf {F}}_{\textrm{M}}$ is a 2-nd order tensor and is symmetric. The parameter range for $({\textbf {F}}_\textrm{M} - {\textbf {I}}_2, \lambda )$ is $\mathbb {M} = [-0.05, 0.05]^3 \times [0.2, 5]$. Building on the concepts of the TTDN method, we obtain the function of $\textrm{NN}_t$, which is given by $g_{t}: ({\textbf {F}}_{\textrm{M}}, \lambda ) \mapsto \varvec{\theta }^{\textrm{RB}}$, where $\varvec{\theta }^{\textrm{RB}}$ are the coefficients of the RB $\{\xi _p^{\textrm{RB}}\}_{p=1}^{Q^\textrm{r}}$.

In this example, we only implement the micro-scale simulation, and the most important quantity to be determined is ${\textbf {K}}_\textrm{eff}$. The quantity ${\textbf {P}}_{\textrm{m}}$ is also of interest since it decides what ${\textbf {K}}_{\textrm{eff}}$ will be. In the sequel, we avoid using the subscript “$\textrm{m}$” to simplify notation. As shown in Fig. 16, the mesh has 5903 nodes.

The non-linearity of the PDE is dependent on the sophisticated strain energy $\mathcal {E}$ that also decides the constitutive law $\mathcal {C}: ({\varvec{{u}}}, \lambda ) \mapsto {\textbf {P}}$. Hence, the data, i.e., $\Xi _P$, of the constitutive law is not as inexpensive as it was in the numerical example discussed in Subsect. “Non-linear heat transfer problem”. In the context of this numerical experiment, we observe that the evaluation of one point for $\Xi _P$ is 24 times cheaper than one full-order solution for $\Xi _U$. Hence, we are not able to generate large enough constitutive data $\Xi _P$ to get the “good enough” pretrained $\textrm{NN}_p$. We thus only train TTDN via the first option as discussed in Sect. “Connection to existing methods”.

We implement the micro-scale simulation in both P1 and P2 elements for the full order Finite Element solver to mimic the problems with full order solutions in different computational cost, which has the impact of the efficiency of the proposed TTDN method.

**Table 9:** Network architecture

**Network**	**Input**	**Intermediate**			**Output**
$\textrm{NN}_u^{\textrm{pdnn}}$	4 StdLy	$4 \rightarrow 60$ StdLy	60 ResNet	80 ResNet	23 StdLy
	1	1	8 if $M_U = 200, 400$ 0 if $M_U = 800, 1500$	0 if $M_U = 200, 400$ 8 if $M_U = 800, 1500$	1
$\textrm{NN}_p^{\textrm{pdnn}}$	4 StdLy	$4 \rightarrow 60$ StdLy	60 ResNet	80 ResNet	45 StdLy
	1	1	10 if $M_U = 200, 400$ 0 if $M_U = 800, 1500$	0 if $M_U = 200, 400$ 10 if $M_U = 800, 1500$	1
$\textrm{NN}_u$	4 StdLy	$4 \rightarrow 60$ StdLy	60 ResNet	80 ResNet	23 StdLy
	1	1	8 if $M_U = 200, 400$ 0 if $M_U = 800, 1500$	0 if $M_U = 200, 400$ 8 if $M_U = 800, 1500$	1
$\textrm{NN}_p$	27 StdLy	$27 \rightarrow 100$ StdLy	100 ResNet		45 StdLy
	1	1	4 if $M_U = 200$; 6 if $M_U = 400$; 8 if $M_U = 800$; 10 if $M_U = 1500$		1

*Numerical setting (with P2 elements):* We use the P2 elements for the 2D vector ${\varvec{{u}}}$ and dP1 elements for the $2 \times 2$ tensor ${\textbf {P}}$. The size of the reduced basis is set to be $N^{\textrm{r}} = 23$ for ${\varvec{{u}}}$ and $Q^\textrm{r} = 45$ for ${\textbf {P}}$, corresponding to an average projection error of ${10^{-3}}$. Our main objective is to show the advantages and disadvantages of the proposed TTDN method by comparing the approximation accuracy of the PDNN and the TTDN method (in accordance with option *(i)* discussed in Subsection Connection to existing methods) with different sizes of full-order training data $\Xi _U$ and different sizes of constitutive law data $\Xi _P$. In this numerical experiment, we choose 4 different sizes $M_U \in \{200, 400, 800, 1500\}$ of the full-order training data $\Xi _U$. For each $M_U$, we choose 3 different sizes $M_P \in \{5M_U, 8M_U, 10M_U\}$ of the constitutive training data $\Xi _P$ to pretrain $\textrm{NN}_p$ of the TTDN method. In the following, the behavior corresponding to the three different sizes of $M_P$ is denoted by TTDN-5, TTDN-8, and TTDN-10, respectively. A full-order training size of 1500 seems to be far from “large enough” eventhough the generation of this full-order data is already so expensive — it takes around $\{7000\}$s to generate the full-order data.

All the networks for the PDNN and the TTDN method have similar architectures with different depths and widths that depend on the size of the training data. The reason is that, with a large training data, we are able to use a large network with large capacity that may suffer from overfitting if the training data is small. The architectures are detailed in Table 9.

**Fig. 18:**
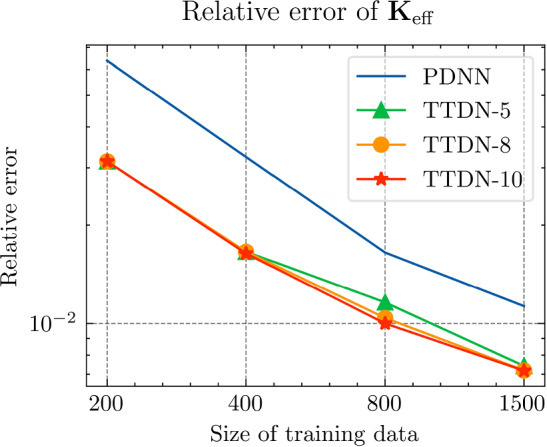
The generalization relative error of ${\varvec{K}}_{\textrm{eff}}$ by different methods and training data

**Fig. 19:**
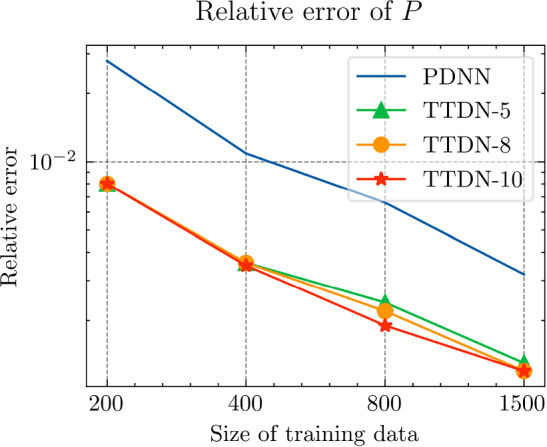
The generalization relative error of ${\varvec{P}}$ by different methods and training data

**Fig. 20:**
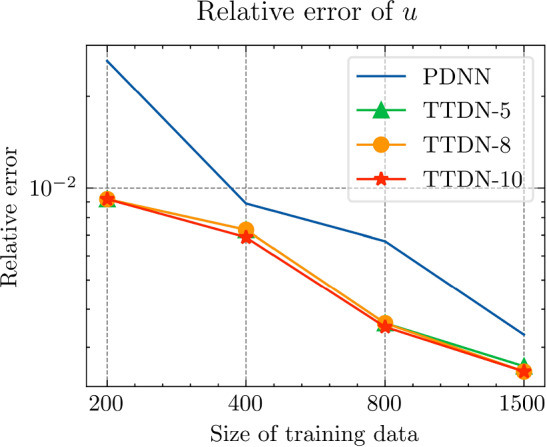
The generalization relative error of ${\varvec{{u}}}$ by different methods and training data

**Fig. 21:**
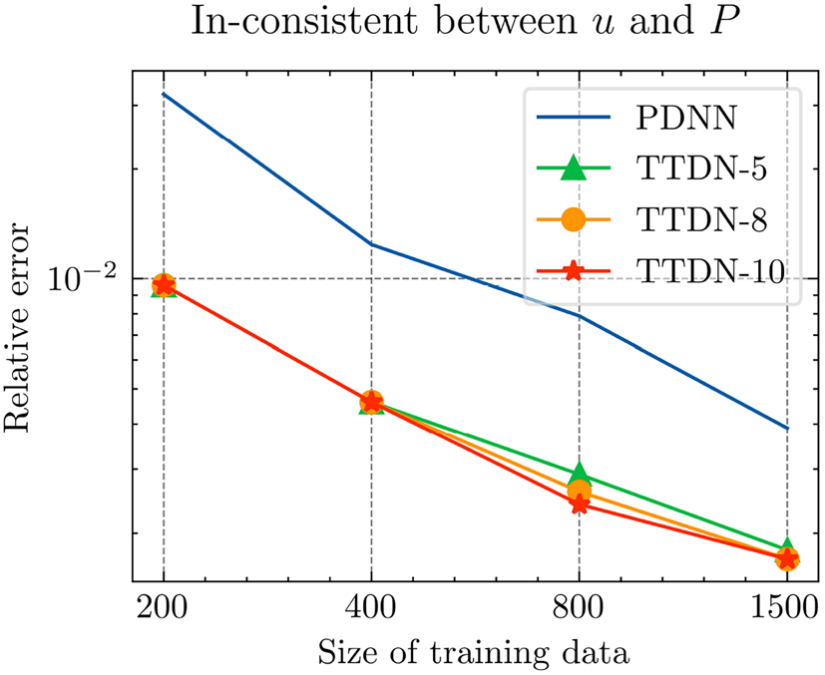
The inconsistency between ${\varvec{{u}}}$ and ${\varvec{P}}$ by different methods and training data

*Numerical observations (with P2 elements):* The comparison of the output effective stiffness tensor ${\textbf {K}}_\textrm{eff}$, the micro-scale stress ${\textbf {P}}$, the micro-scale intermediate displacement ${\varvec{{u}}}$ (which is denoted by $\tilde{{\varvec{{u}}}}$ in the above description) and, finally, the inconsistency between ${\varvec{{u}}}$ and ${\textbf {P}}$, is shown in Figs. 18–21. These figures show the average relative generalization errors of the physical quantities for 300 arbitrarily chosen parameters. In Figs. 18–21, we observe that, when $M_U=200$, the PDNN method does not perform well. The errors of both ${\textbf {P}}$ and ${\varvec{{u}}}$ are larger than ${10^{-2}}$, and the error of ${\textbf {K}}_{\textrm{eff}}$ is even larger than $5 \times 10^{-2}$. In this case, the small training data limits the capacity of the generalization of the PDNN method. However, the TTDN method improves this capacity by introducing the second tier network that learns the constitutive training data. In this case, the resulting errors in ${\textbf {P}}$ and ${\varvec{{u}}}$ are below ${10^{-2}}$. In fact, as shown in the aforementioned figures, the performance of the TTDN method with different sizes of the training data $\Xi _U$ of $M_U$ is always comparable to the performance of the PDNN method with the size of the training data $\Xi _U$ of $2M_U$. Furthermore, the former outperforms the latter for the derived quantity ${\textbf {P}}$.

**Fig. 22:**
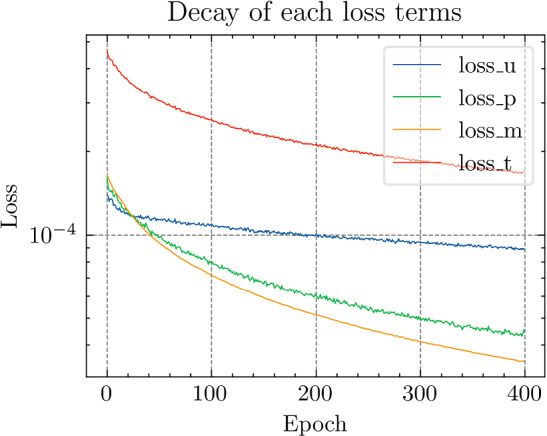
Loss curves for the different terms

We also observe that the increase of $M_P$, i.e., the size of the constitutive training data, does not seem to improve the performance of the TTDN method. As already mentioned, the evaluation of the constitutive law is not much cheaper than the full-order solution, and we are not able to generate very large $\Xi _P$. This means that the second-tier $\textrm{NN}_p$ cannot approximate the constitutive law with a very high accuracy, and that we have to use the the original version of TTDN. During the semi-supervised learning of $\textrm{NN}_t$, we find that the training error of $\Xi _U$ dominates the loss function, see Fig. 22 for the curves of each loss terms. This is also revealed by the fact that the generalization error of ${\varvec{{u}}}$ is larger than ${\textbf {P}}$, see Figs. 19 and 20. As a consequence, the misfit by $\Xi _U$ contributes more to the gradient descent step than $\Xi _P$. In other words, the accuracy of ${\varvec{{u}}}$ is the bottleneck. Conversely, the loss associated with $\Xi _P$ contributes less during the training stage and, hence, increasing $M_P$ does not provide large improvements to the results obtained using the TTDN method.

From Figs. 19 and 20, we can observe that, using the first training option, the TTDN method improves the accuracy of ${\textbf {P}}$ more than that of ${\varvec{{u}}}$. This behavior can be explained using the curves in Fig. 22, which show that the reduction of the loss of ${\textbf {P}}$ is larger than that of *u*. Consequently, the accuracy of ${\textbf {P}}$ obtained using the TTDN method is obviously higher than the one by the PDNN method; see Fig. 19. We should also notice that, as the average projection error of ${\varvec{{u}}}$ and ${\textbf {P}}$ is ${10^{-3}}$, the accuracy of ${\textbf {P}}$, computed using the TTDN method, is very close to this best achievable error, i.e., ${10^{-3}}$, when $M_U=1500$.

However, it seems that the accuracy of ${\textbf {K}}_{\textrm{eff}}$ does not take advantages of this effect—the accuracy of ${\textbf {K}}_{\textrm{eff}}$ in Fig. 18 by the TTDN method is improved, but not as much as the accuracy of ${\textbf {P}}$. We are aware of the fact that the entire TTDN, i.e., $\textrm{NN}_t$, is very deep. We choose to reduce the depth of $\textrm{NN}_p$ when the size of $\Xi _P$ is reduced. As a small experiment, we have done a test for the TTDN setting of $M_U=800$ and $M_P=10M_U$ with 10 layers of ResNet. In this case, we get a better accuracy of ${\textbf {P}}$, i.e., around $\frac{2}{3}$ of the error of the current setting that contains 8 layers. However, the accuracy of ${\textbf {K}}_{\textrm{eff}}$ is worse (increases by $\sim 15\%$) with more number of ResNet layers. This behavior can be argued from the fact that, in deep learning, while the depth improves the capacity to learn the non-linearity, it also makes the learned function become less smooth and, thus, reduce the accuracy of the derivative, i.e., ${\textbf {K}}_{\textrm{eff}}$, see [68, 85].

**Fig. 23:**
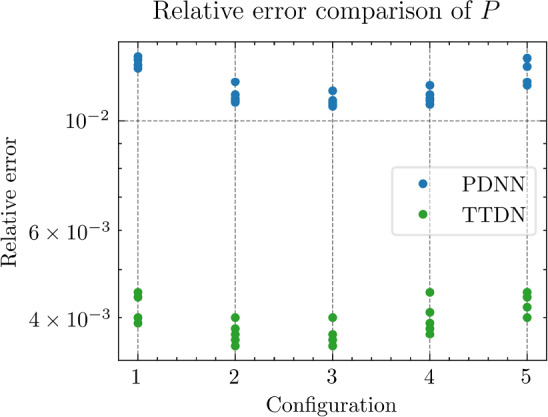
Generalization error for different hyper-parameter settings and initialization, see Table 10

**Table 10:** Learning rate setting for each configuration

**Config.**	**1**	**2**	**3**	**4**	**5**
PDNN	${5\times 10^{-3}}$	${2\times 10^{-3}}$	${10^{-3}}$	${8\times 10^{-4}}$	${5\times 10^{-4}}$
TTDN	${5\times 10^{-5}}$	${4\times 10^{-5}}$	${3\times 10^{-5}}$	${2\times 10^{-5}}$	${\times 10^{-5}}$

The table shows the learning rate for semi-supervised learning for TTDN. The pretrain learning rate of TTDN is set to be ${10^{-3}}$, the best setting as per the results from PDNN

Figure 21 shows the results regarding consistency between ${\varvec{{u}}}$ and ${\textbf {P}}$, which is defined in (23). We see that the proposed TTDN method provides the results that have higher consistency between the unknown quantity ${\varvec{{u}}}$ and the derived quantity ${\textbf {P}}$. This means that the results obtained by the TTDN method are more in line with the constitutive law of the underlying PDE than those obtained by the PDNN method.

Figure 23 presents an investigation of the sensitivity of the performance to the hyper-parameter settings. It shows the relative test error of ${\textbf {P}}$ for different hyper-parameter configurations, shown in Table 10, using the setting of $M_U = 400$, $M_P = 5 M_U$. The different points in green or blue for each configuration correspond to different random seeds used for Gaussian initialization of the NN trainable parameter. According to this figure, the “best” learning rate for semi-supervised learning is roughly 2 orders of magnitude less than the learning rate for pretraining TTDN and training PDNN. This can be explained from the fact that the network for semi-supervised learning is pretrained and only needs fine tuning. This “best” learning rate is used in generating the figures shown in this subsection. However, it is worth mentioning that, based on the numerical results, the accuracy of these methods is relatively insensitive to the learning rate and the Gaussian initialization. In addition, in our numerical experiments, the influence of other hyper-parameters, e.g., the size of a mini-batch, if applying dropout, to the final predictive accuracy seems to be insignificant.

**Fig. 24:**
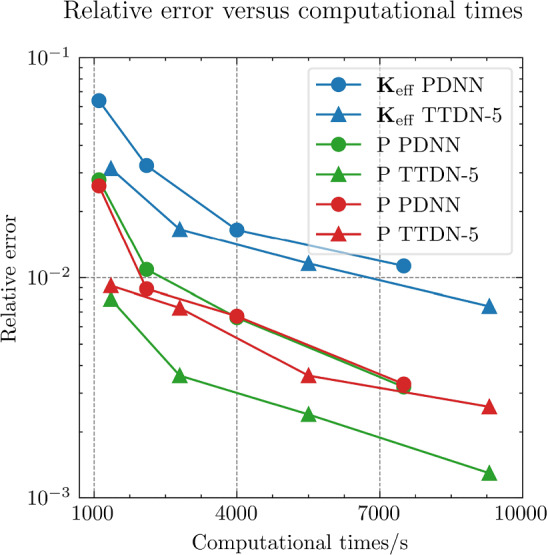
Computational time that needed for different relative error for PDNN and TTDN method

**Fig. 25:**
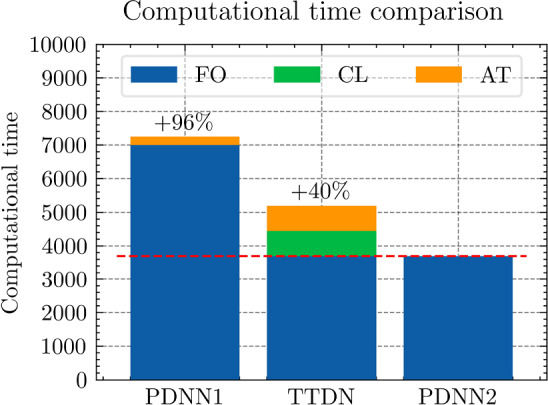
The comparison of computational time (in seconds) for PDNN and TTDN. Here, FO stands for means full-order, data CL is the data of the constitutive law, and AT is the additional training time over PDNN2

**Fig. 26:**
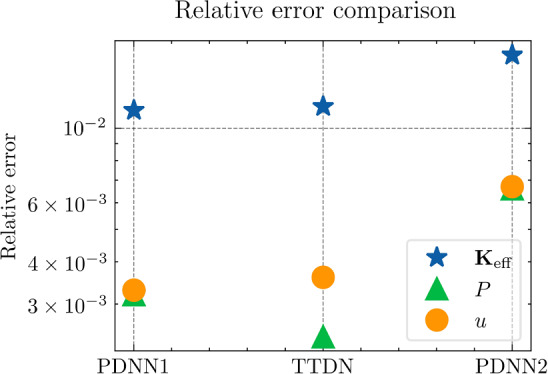
The comparison of the generalization accuracy of ${\varvec{K}}_\textrm{eff}$, ${\varvec{P}}$ and ${\varvec{{u}}}$. Detailed settings of PDNN and TTDN are shown in the discussion

*Computational costs (with P2 elements):* Finally, we will discuss the computational costs of the PDNN and the proposed TTDN method. As mentioned earlier, the computational time for generating 1500 full-order solutions and 15000 evaluations of the constitutive law is around 7000s and 2800s, respectively. As for the different sizes of the training data, the required computational time is different. Fig. 24 presents the achieved accuracies of *u*, ${\textbf {P}}$ and ${\textbf {K}}_{\textrm{eff}}$ for PDNN and TTDN, with respect to the computational time (which includes the time for training data generation and the time for training). In this figure, the more the curve tends to the lower-left, the better the performance of the method.

Specifically, we compare the setting of $M_U=800$, $M_P=4000$ for the TTDN method (denoted by TTDN) to the setting of $M_U=1500$ for the PDNN method (denoted by PDNN1) since they have similar performance, and to the setting of $M_U=800$ for the PDNN method (denoted by PDNN2) since they have the same $M_U$; see Figs. 25–26. The comparison of other sizes of full-order training data is similar. Please note that, for TTDN, we need additional time compared to PDNN2 to train the network $\textrm{NN}_t$ in a semi-supervised manner. This is a drawback of the proposed method. Furthermore, as the data size $M_U$ increases, PDNN1 needs more time to train than PDNN2. The training time for neural network is non-deterministic as it is a randomized algorithm due to an arbitrary initial state and the optimizer of the stochastic gradient decent method and, hence, we only provide a rough additional time. This, for TTDN and PDNN1, respectively, amounts to around 750s and around 250s over PDNN2; see Fig. 25. All the networks in this work are trained using CPU, which is believed to be inefficient for NN training. One can expect to have a large improvement with other devices, e.g., powerful GPGPUs like CUDA$^\circledR$, ROCm™, etc.

In Figs. 25–26, we see that, to improve the accuracy from PDNN2 to PDNN1, using the PDNN method, we need to pay $96\%$ more computational time. However, by the proposed TTDN method, we need only $40\%$ additional time to attain the similar accuracy as that of PDNN1. If the full-order problem is more complicated, i.e., the computational cost for the full-order solution is higher, then the difference of the computational time of the two methods, i.e., PDNN and TDNN, will become more obvious. In particular, if we compare the derived quantity ${\textbf {P}}$, in this setting, TTDN has higher accuracy than PDNN1. Hence, the proposed TTDN method can improve the generalization accuracy of the neural network in conjunction with MOR with limited expensive full-order training data. However, in this example, since the size of the constitutive law data $\Xi _P$ is also limited—only 5 to 10 times of $\Xi _U$, the second layer of TTDN, $\textrm{NN}_p$, is not so accurate. In this case, the proposed method cannot give a large improvement for the unknown quantity ${\varvec{{u}}}$, but can still improve the accuracy of the derived quantity ${\textbf {P}}$ by a large amount.

**Fig. 27:**
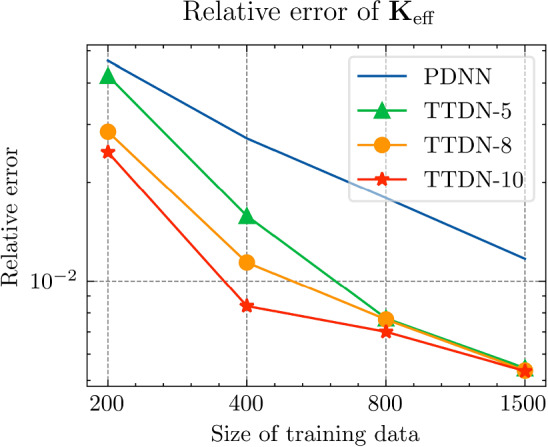
The generalization relative error of ${\varvec{K}}_{\textrm{eff}}$ by different methods and training data, P1 setting

**Fig. 28:**
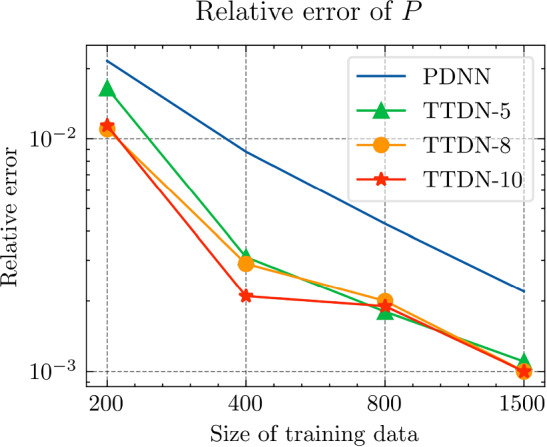
The generalization relative error of ${\varvec{P}}$ by different methods and training data, P1 setting

**Fig. 29:**
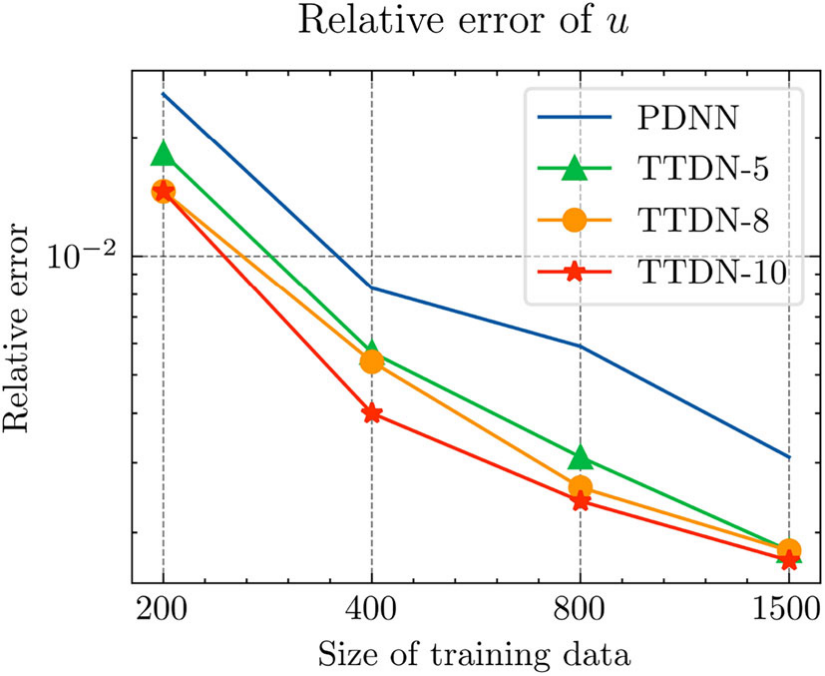
The generalization relative error of ${\varvec{{u}}}$ by different methods and training data, P1 setting

**Fig. 30:**
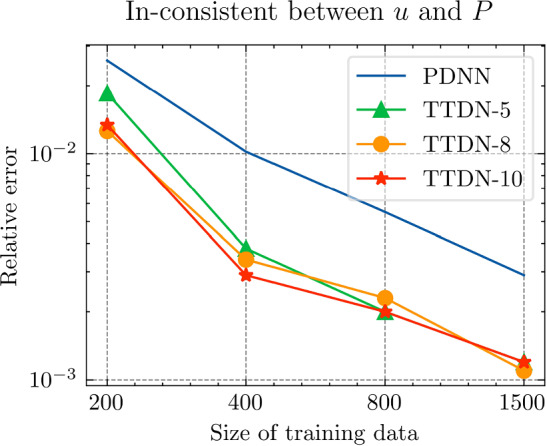
The inconsistent between ${\varvec{{u}}}$ and ${\varvec{P}}$ by different methods and training data, P1 setting

*Numerical setting (with P1 elements):* Next, we implement the same problem setting with P1 finite elements to test the performance of the TTDN method in the case when the computation of full-order solutions is relatively inexpensive. Please note that the training cost does not decrease since the network and the training data have a similar size. Only the computational cost of the full-order solution and the evaluation of the constitutive law that are in the stage of the training data generation are reduced. In this case, all relative errors are also compared to the results of the P1 setting. With the same average relative projection error ${10^{-3}}$, the dimension of the reduced basis of ${\varvec{{u}}}$ and ${\textbf {P}}$ is, respectively, $N^{\textrm{r}} = 26$, $Q^{\textrm{r}} = 54$. It is clear that the dimensions are slightly greater than those for the P2 setting. We use the same settings of the network for P2 setting, shown in Table 9, except the dimensions of the input and the output that are decided by $N^{\textrm{r}}$ and $Q^\textrm{r}$. The results are shown in Figs. 27, 28, 29 and 30.

**Fig. 31:**
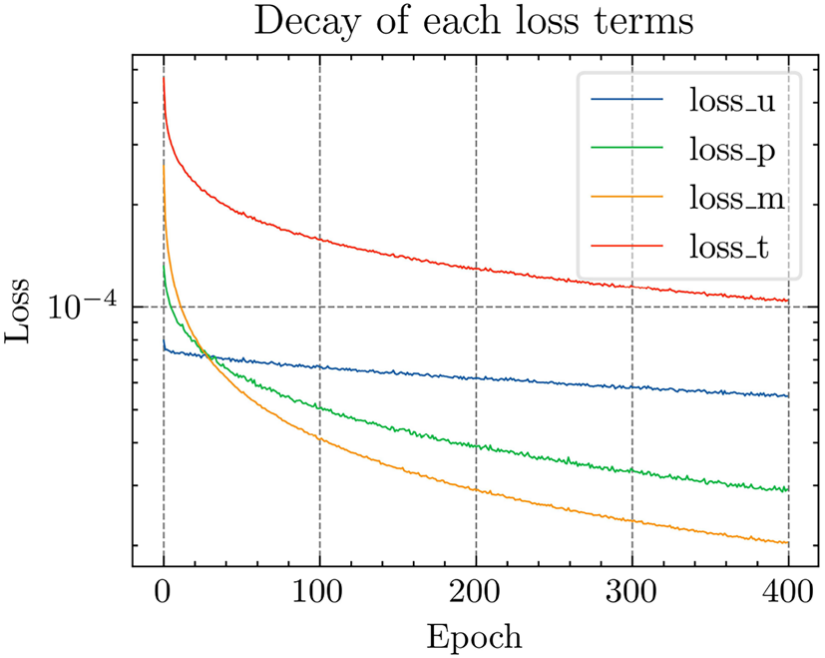
Loss curves for the different terms, P1 setting

*Numerical observations (with P1 elements):* We observe that the increase of $M_P$ can slightly improve the prediction, but still not in an obvious way. In this setting, compared to the P2, the difference between the accuracy of ${\textbf {P}}$ and ${\varvec{{u}}}$ is reduced. Hence, it places less importance to the misfit of $\Xi _U$ during semi-supervised learning, making the loss associated with $\Xi _P$ contribute more during the training. See Fig. 31 for the loss terms corresponding to $\Xi _U$ and $\Xi _P$. Due to this effect, increasing the size of $\Xi _P$ can help to improve the predictive accuracy. This effect is, however, not too obvious in the numerical experiments. The slightly higher accuracy of ${\varvec{{u}}}$ compared to that with the P2 setting may be due to the stochastic nature of ML techniques and also due to the effect of parameter sharing in the domain of ML. We will discuss this effect below.

We see that the difference of accuracy from the P2 element setting also happens in the accuracy of ${\textbf {K}}_\textrm{eff}$. Recall that the number of the output features in this setting is greater than that in the P2 setting. Counterintuitively, due to the effect of parameter sharing, the higher the dimensionality of the input and output, the less is the overfitting risk in the case where the output features have underlying relation(s); we refer the reader to Chapter 7 in [28] for more details. In fact, the trainable parameters are shared for all the output channels, meaning that the training data at all the output channels are helpful in learning the optimum trainable parameters. When the number of trainable parameters remain unchanged, increasing the dimensionality of the output will also enrich the available information in each training data point and, hence, provide richer information to prevent overfitting, which is harmful especially when the derivative of the network is sought since it makes the map of the network less smooth. Hence, compared to the P2 setting, TTDN for the P1 setting has larger input and output dimensionality, which ultimately leads to better improvement in accuracy of *u* and ${\textbf {K}}_{\textrm{eff}}$.

**Fig. 32:**
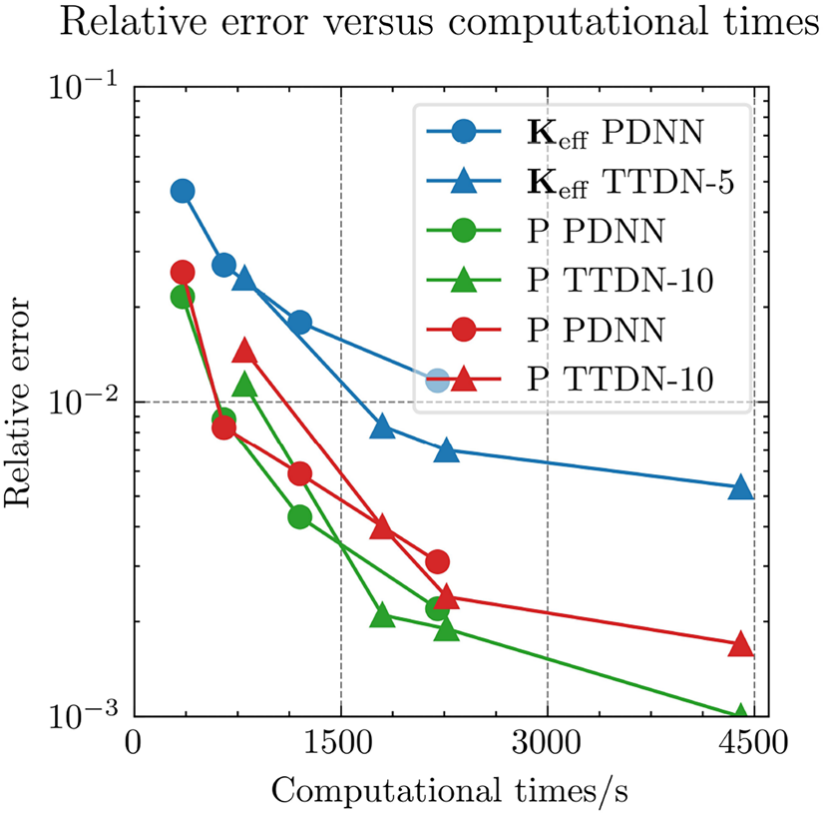
Computational time for different relative error for PDNN and TTDN method, P1 setting

**Fig. 33:**
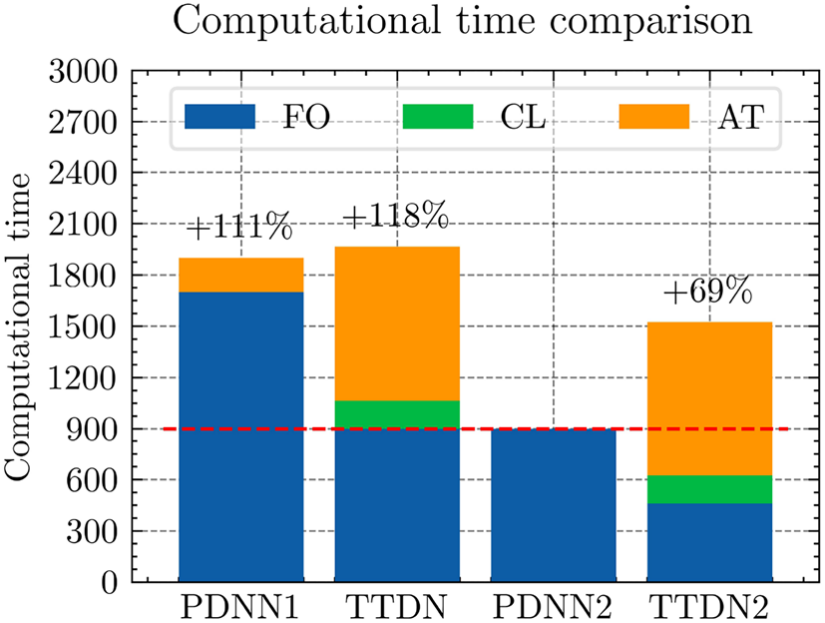
The comparison of computational time (in seconds) for PDNN and TTDN for P1 setting. Here, FO stands for full-order data, CL is the data of the constitutive law, and AT is the additional training time over PDNN2

**Fig. 34:**
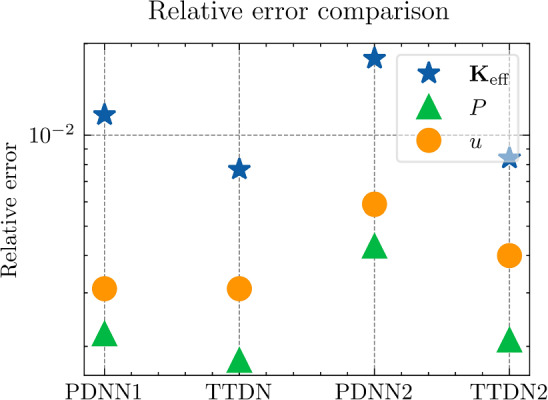
The comparison of the generalization accuracy of ${\varvec{K}}_\textrm{eff}$, ${\varvec{P}}$ and ${\varvec{{u}}}$ for P1 setting. Detailed settings of PDNN and TTDN are show in the discussion

*Computational costs (with P1 elements):* For the computational time, the situation is quite different since, in this case, the cost for data generation is quite inexpensive compared to the P2 setting and hence, the additional training time for the TTDN method can be a factor that makes it inefficient. See Fig. 32 for the curves of achieved accuracy versus the required computational time. Overall, we can see that TTDN does not outperform PDNN. Precisely, we shift to Figs. 33-34, where the setting of PDNN1, TTDN and PDNN2 are the same as the settings discussed above for the P2 setting. In this problem, as the unknown ${\varvec{{u}}}$ and the output quantity ${\textbf {K}}_{\textrm{eff}}$ have higher accuracy, we also draw the comparison to the setting TTDN2 with $M_U = 400$ and $M_P = 10M_U$. We can see that TTDN2 has accuracy similar to that of PDNN1. However, since the full-order solution is not expensive, the training time for $\textrm{NN}_t$ can be comparable to the time for the generation of the full-order data; see Fig. 33, wherein it can be observed that the additional time of TTDN over PDNN2 is even higher than that of PDNN1 since the training takes too much time, even though TTDN has higher accuracy for ${\textbf {K}}_{\textrm{eff}}$. Even for the setting of TTDN2 that feeds less full-order data $\Xi _U$, the efficiency is not as good as that for the P2 setting since the time saved in generating full-order solutions is offset by the additional training time.

By this comparison, we see another disadvantage of the proposed TTDN method: we need to pay the price of additional training time, which can be relatively large when the full-order solution is not expensive. One may overcome this difficulty by implementing and training the network in an efficient GPU. It, however, requires additional specialized hardware.

## Conclusions and outlook

In this work, we proposed a novel physics-informed machine learning method—the TTDN method. The network is formed by the combination of two tiers—the first tier achieves the regression of the unknown quantity of interest, while the second tier rebuilds the physical constitutive law of the underlying partial differential equation between the unknown quantity of interest *u* and the derived quantity *P*. To summarize, the advantages of the proposed TTDN method are listed below:

- With limited full-order training data, which is obtained by full-order solutions that are expensive in general, the proposed method is able to provide more accurate approximations for both the unknown quantity of interest *u* and derived quantity *P*.
- The obtained predictions are physically consistent compared to the ones resulting from the conventional PDNN.
- The second tier network catches the physical laws and can provide more underlying physical information, e.g., $\frac{\partial P}{\partial u}$ can be obtained by a back-propagation of $\textrm{NN}_p$.
- The second tier of the network, $\textrm{NN}_p$, also provides a tool to achieve general hyper-reduction. The numerical tests show that this method has a higher efficiency compared to some other hyper-reduction tool methods, e.g., (D)EIM.
- As a non-intrusive method, it is relatively straightforward to implement.

The proposed method, however, also has the following disadvantages:

- When the full-order training data is already large enough, the generalization error of the direct regression method, e.g., the PDNN, can be small. In this case, the proposed method may perform worse than the PDNN.
- When the evaluation of the constitutive law is not much cheaper than the the full-order solving, we are unable to generate large constitutive data, and TTDN may not be able to achieve a significant improvement in terms of offline time.
- The training of $\textrm{NN}_t$, compared to the conventional PDNN, needs additional time for the extra unsupervised learning stage. If the full-order solution is not expensive, and the training of the network is not efficient, then the additional time for training may match or even exceed the time saved by a smaller full-order data.

The proposed method may also work with the recurrent neural network and has the potential to work for a dynamical system. For this application, there may be two possible options for the architecture of the recurrent network: *(i)* the circuit block contains $\textrm{NN}_u$ and $\textrm{NN}_p$; *(ii)* a fixed tier of $\textrm{NN}_p$ by feed-forward network works with the recurrent network that contains $\textrm{NN}_u$ as the circuit block. This will be a subject of future investigation.

## Data Availability

All data generated or analysed during this study are included in this published article. The relevant codes for the proposed method are available in the TTDN repository, https://github.com/Yankun-Hong/TTDN.

## Appendix A: Definitions of matrices and vectors

The definitions of the Finite Element matrix and vectors introduced in Subsect. “Full-order model” are provided here:

- ${\textbf {M}}^{\textrm{FE}} \in \mathbb {R}^{N^{\textrm{f}} \times N^{\textrm{f}}}$ is defined by $[{\textbf {M}}^{\textrm{FE}}(\tilde{{\textbf {u}}}^{\textrm{FE}}; \varvec{\mu })]_{j,i} = - \left\langle \mathcal {A} P^{\prime }({u}^{\textrm{f}}, \phi _i^{\textrm{FE}}; \varvec{\mu }), \phi _j^{\textrm{FE}} \right\rangle$,
- ${\textbf {f}}^{\textrm{FE}} \in \mathbb {R}^{N^{\textrm{f}}}$ is defined by $({\textbf {f}}^{\textrm{FE}})_j = f(\phi _j^{\textrm{FE}})$,
- ${\textbf {p}}^{\textrm{FE}} \in \mathbb {R}^{N^{\textrm{f}}}$ is defined by $({\textbf {p}}^{\textrm{FE}}(\tilde{{\textbf {u}}}^{\textrm{FE}}; \varvec{\mu }))_{j} = \left\langle \mathcal {A} P(\tilde{u}^{\textrm{f}}; \varvec{\mu }), \phi _{j}^{\textrm{FE}}\right\rangle$.

For more details, refer to some Finite Element Method books, e.g., [1].

The definition of the Reduced Basis matrix and vectors introduced in Subsection “Reduced basis method” are provided here:

- ${\textbf {M}}^{\textrm{RB}} \in \mathbb {R}^{N^{\textrm{r}} \times N^{\textrm{r}}}$ is defined by $[{\textbf {M}}^{\textrm{RB}}(\tilde{{\textbf {u}}}^{\textrm{RB}}; \varvec{\mu })]_{j,i} = - \left\langle \mathcal {A} P^\prime (\tilde{u}^{\textrm{r}}, \phi _i^{\textrm{RB}}; \varvec{\mu }), \phi _j^{\textrm{RB}} \right\rangle$,
- ${\textbf {f}}^{\textrm{RB}}$ is defined by $({\textbf {f}}^{\textrm{RB}})_j = f(\phi _j^{\textrm{RB}})$,
- ${\textbf {p}}^{\textrm{RB}} \in \mathbb {R}^{N^{\textrm{r}}}$ is defined by $({\textbf {p}}^{\textrm{RB}}(\tilde{{\textbf {u}}}^{\textrm{RB}}; \varvec{\mu }))_j = \left\langle \mathcal {A} P(\tilde{u}^{\textrm{r}}; \varvec{\mu }), \phi _j^{\textrm{RB}}\right\rangle$,
- ${\textbf {B}} \in \mathbb {R}^{N^{\textrm{r}} \times N^{\textrm{f}}}$ is defined by $[{\textbf {B}}]_{i,j} = b_{ji}$, where $b_{ji}$ is the coefficient such that $\phi _i^{\textrm{RB}} = \sum _{j=1}^{N^{\textrm{f}}} b_{ji} \phi _j^{\textrm{FE}}$.

For more details, refer to some Reduced Basis Method books, e.g., [4, 5].

The definition of the hyper-reduction matrices and vectors introduced in Subsect. “General hyper-reduction” are provided next.

Using (13), we have the expansions $\left\langle \mathcal {A} P^\prime (\tilde{u}^{\textrm{r}}, \phi _i^{\textrm{RB}}; \varvec{\mu }), \phi _j^{\textrm{RB}} \right\rangle \approx \sum _{p = 1}^{Q^{\textrm{r}}} \left. \frac{\partial ({\textbf {P}}^{\textrm{HR}}({\textbf {u}}^{\textrm{RB}}; \varvec{\mu }))_p}{\partial ({\textbf {u}}^\textrm{RB})_i}\right| _{{\textbf {u}}^{\textrm{RB}}=\tilde{{\textbf {u}}}^{\textrm{RB}}} \cdot \left\langle \mathcal {A} \xi _p^{\textrm{RB}}, \phi _j^{\textrm{RB}} \right\rangle$ as well as $\left\langle \mathcal {A} P(\tilde{u}^{\textrm{r}}; \varvec{\mu }), \phi _j^{\textrm{RB}}\right\rangle \approx \sum _{p = 1}^{Q^{\textrm{r}}} ({\textbf {P}}^{\textrm{HR}}(\tilde{{\textbf {u}}}^{\textrm{RB}}; \varvec{\mu }))_p \cdot \left\langle \mathcal {A} \xi _p^{\textrm{RB}}, \phi _j^{\textrm{RB}} \right\rangle$. This leads to the matrices

- ${\textbf {M}}_c \in \mathbb {R}^{N^{\textrm{f}} \times Q^{\textrm{f}}}$, which is defined by $[{\textbf {M}}_c]_{j,q} = \left\langle \mathcal {A}\xi _q^{\textrm{FE}}, \phi _j^{\textrm{FE}}\right\rangle$,
- ${\textbf {C}}_{\textrm{hr}} \in \mathbb {R}^{Q^{\textrm{r}} \times Q^{\textrm{f}}}$, which is defined by $[{\textbf {C}}_{\textrm{hr}}]_{p,q} = c_{qp}$, where $c_{qp}$ denotes the coefficient such that $\xi ^{\textrm{RB}}_p = \sum _{q=1}^{Q^{\textrm{f}}} c_{qp} \xi _q^{\textrm{FE}}$ and is computed by the general hyper-reduction methods,
- ${\textbf {J}}^{\textrm{HR}} \in \mathbb {R}^{Q^{\textrm{r}} \times N^{\textrm{r}}}$, which is defined by $[{\textbf {J}}^{\textrm{HR}}]_{p,i} = \left. \frac{\partial ({\textbf {P}}^{\textrm{HR}})_p}{\partial ({\textbf {u}}^{\textrm{RB}})_i}\right| _{{\textbf {u}}^{\textrm{RB}}=\tilde{{\textbf {u}}}^{\textrm{RB}}}$.

For more details, refer to some Reduced Basis Method books, e.g., [4].

## Appendix B: Supplement to Sect. “Non-linear heat transfer problem”

We approximate the unknown *u* by the coefficient vector ${\textbf {u}}$ and the reduced basis $\{\phi _i^\textrm{RB}\}_{i=1}^{N^{\textrm{r}}}$ as ${\varvec{{u}}}({\varvec{{x}}}) = \sum _{i = 1}^{N^{\textrm{r}}} ({\textbf {u}})_i \phi _i^{\textrm{RB}}({\varvec{{x}}})$. Similarly, we obtain the approximation to the derived quantity ${\varvec{{q}}}$ and its derivative by the basis $\{\xi _p^\textrm{RB}\}_{p=1}^{Q^{\textrm{r}}}$ and coefficients $\{\theta _p\}_{p=1}^{Q^{\textrm{r}}}$:

Baa $$\begin{aligned} {\varvec{{q}}}({\textbf {u}}, \bar{\kappa })&= \sum _{p = 1}^{Q^{\textrm{r}}} \theta _p({\textbf {u}}, \bar{\kappa }) \xi _p^{\textrm{RB}}({\varvec{{x}}}), \end{aligned}$$

Bab $$\begin{aligned} \frac{\partial {\varvec{{q}}}}{\partial {\textbf {u}}}({\textbf {u}}, \bar{\kappa })&= \sum _{p = 1}^{Q^{\textrm{r}}} \frac{\partial \theta _p}{\partial {\textbf {u}}}({\textbf {u}}, \bar{\kappa }) \xi _p^{\textrm{RB}}({\varvec{{x}}}). \end{aligned}$$

At the *n*-th Newton iteration step, with the current state denoted by ${\textbf {u}}^{n-1}$, by differentiating the left hand side of (22) and plugging in (Ba), the correction $(\delta {\textbf {u}}^n)_i$ satisfies, for all $j = 1, ..., N^{\textrm{r}}$,

$$\begin{aligned} \sum _{p = 1}^{Q^{\textrm{r}}} \int _{\Omega }&{\xi _p^{\textrm{RB}}} \cdot \nabla \phi _j^{\textrm{RB}} \, \textrm{d}V \sum _{i=1}^{N^{\textrm{r}}} \frac{\partial \theta _p}{\partial ({\textbf {u}})_i} (\delta {\textbf {u}}^n)_i - h \sum _{i = 1}^{N^{\textrm{r}}} \int _{\Gamma _{\textrm{r}}} \phi _i^{\textrm{RB}} \phi _j^{\textrm{RB}} \, \textrm{d}S \, (\delta {\textbf {u}}^n)_i = \\&- h u_{\textrm{env}} \int _{\Gamma _{\textrm{r}}} \phi _j^{\textrm{RB}} \,\textrm{d}S - q_{\Gamma } \int _{\Gamma _{\textrm{n}}} \phi _j^{\textrm{RB}} \,\textrm{d}S + h \sum _{i=1}^{N^{\textrm{r}}}({\textbf {u}}^{n-1})_i \int _{\Gamma _{\textrm{r}}} \phi _i^{\textrm{RB}} \phi _j^{\textrm{RB}} \,\textrm{d}S \\&- \sum _{p = 1}^{Q^{\textrm{r}}} \theta _p({\textbf {u}}^{n-1}) \int _{\Omega } \xi _p^{\textrm{RB}} \cdot \nabla \phi _j^{\textrm{RB}} \, \textrm{d}V. \end{aligned}$$

For the matrix formulation, we compute, offline, the quantities ${\textbf {M}}_{u} \in \mathbb {R}^{N^{\textrm{r}} \times N^\textrm{r}}$, ${\textbf {M}}_{c} \in \mathbb {R}^{N^{\textrm{r}} \times {Q^\textrm{r}}}$, ${\varvec{{f}}}^1 \in \mathbb {R}^{N^{\textrm{r}}}$, ${\varvec{{f}}}^2 \in \mathbb {R}^{N^{\textrm{r}}}$ given by

$$\begin{aligned}&[{\textbf {M}}_{u}]_{j,i} = \int _{\Gamma _{\textrm{r}}} \phi _i^{\textrm{RB}} \phi _j^{\textrm{RB}} \,\textrm{d}S,&({\varvec{{f}}}^1)_j = \int _{\Gamma _{\textrm{r}}} \phi _j^{\textrm{RB}} \,\textrm{d}S, \\&[{\textbf {M}}_{c}]_{j,p} = \int _{\Omega } \xi _p^{\textrm{RB}} \cdot \nabla \phi _j^{\textrm{RB}} \, \textrm{d}V,&({\varvec{{f}}}\,^2)_j = \int _{\Gamma _{\textrm{n}}} \phi _j^{\textrm{RB}} \,\textrm{d}S. \end{aligned}$$

In addition, we also train . In the online stage, we reconstruct ${\textbf {J}}_c \in \mathbb {R}^{{Q^{\textrm{r}}} \times N^{\textrm{r}}}$ by $[{\textbf {J}}_c]_{pi} = \frac{\partial \theta _p}{\partial ({\textbf {u}})_i}$, and compute $\delta {\textbf {u}}^n$ for which, at the *n*-th iteration step,

$$\begin{aligned} ({\textbf {M}}_c \cdot {\textbf {J}}_c - h {\textbf {M}}_u) \cdot \delta {\textbf {u}}^n = - h u_{\textrm{env}} {\varvec{{f}}}^1 - q_{\Gamma } {\varvec{{f}}}^2 + h {\textbf {M}}_u \cdot {\textbf {u}}^{n-1} - ({\textbf {M}}_c \cdot \varvec{\theta })({\textbf {u}}^{n-1}) \end{aligned}$$

holds.

## Appendix C: Supplement to Sect. “Time dependent Burgers’ probem”

Using these reduced basis spaces, we obtain the discrete (matrix) representation of the weak formulation:

C4 $$\begin{aligned} {\textbf {M}}_u \cdot \frac{\partial {\textbf {u}}}{\partial t} = - {\textbf {M}}_c \cdot \varvec{\theta }({\textbf {u}}) - \mu _1 {\textbf {M}}_{\nabla } \cdot {\textbf {u}} + \mu _2 {\textbf {F}} \cdot \varvec{\beta }(\mu _3), \end{aligned}$$

where ${\textbf {M}}_{u} \in \mathbb {R}^{N^{\textrm{r}} \times N^{\textrm{r}}}$, ${\textbf {M}}_{c} \in \mathbb {R}^{N^{\textrm{r}} \times Q^\textrm{r}_1}$, ${\textbf {M}}_{\nabla } \in \mathbb {R}^{N^{\textrm{r}} \times N^\textrm{r}}$ and ${\textbf {F}} \in \mathbb {R}^{N^{\textrm{r}}\times Q^{\textrm{r}}_2}$, are given by

$$\begin{aligned}&[{\textbf {M}}_{u}]_{j,i} = \int _{\Gamma _{\textrm{r}}} \phi _i^{\textrm{RB}} \phi _j^{\textrm{RB}} \,\textrm{d}V,&[{\textbf {M}}_{c}]_{j,p} = \int _{\Omega } \xi _p^{\textrm{RB}} \phi _j^{\textrm{RB}} \, \textrm{d}V, \\&[{\textbf {M}}_{\nabla }]_{j,i} = \int _{\Gamma _{\textrm{r}}} \nabla \phi _i^{\textrm{RB}} \cdot \nabla \phi _j^{\textrm{RB}} \,\textrm{d}V,&[{\textbf {F}}]_{j,p} = \int _{\Omega } \zeta _p^{\textrm{RB}} \phi _j^{\textrm{RB}} \,\textrm{d}V. \end{aligned}$$

Note that, for the reduced basis generated by the POD algorithm, ${\textbf {M}}_u = {\textbf {I}}_{N^{\textrm{r}}}$. The Jacobian of $\textrm{NN}_p^1$ is ${\textbf {J}}_c \in \mathbb {R}^{Q^{\textrm{r}}_1 \times N^\textrm{r}}$, which is given by $[{\textbf {J}}_c]_{pi} = \frac{\partial \theta _p}{\partial ({\textbf {u}})_i}$, and the Jacobian of the right-hand side of (C4) is given by

$$\begin{aligned} {\textbf {J}}({\textbf {u}}, \varvec{\mu }) = - {\textbf {M}}_c \cdot {\textbf {J}}_c - \mu _1 {\textbf {M}}_{\nabla }. \end{aligned}$$

Employing the DIRK time discretization scheme, we have the following relations at each time step:

C5a $$\begin{aligned}&{\textbf {M}}_u \cdot {\textbf {u}}_{n,1} = {\textbf {M}}_u \cdot {\textbf {u}}_{n} + \frac{3+\sqrt{3}}{6} {\textbf {f}}({\textbf {u}}_{n,1}) \delta t; \end{aligned}$$

C5b $$\begin{aligned}&{\textbf {M}}_u \cdot {\textbf {u}}_{n,2} = {\textbf {M}}_u \cdot {\textbf {u}}_{n} - \frac{\sqrt{3}}{3} {\textbf {f}}({\textbf {u}}_{n,1}) \delta t + \frac{3+\sqrt{3}}{6} {\textbf {f}}({\textbf {u}}_{n,2}) \delta t; \end{aligned}$$

C5c $$\begin{aligned}&{\textbf {M}}_u \cdot {\textbf {u}}_{n+1} = {\textbf {M}}_u \cdot {\textbf {u}}_{n} + \frac{1}{2} {\textbf {f}}({\textbf {u}}_{n,1}) \delta t + \frac{1}{2} {\textbf {f}}({\textbf {u}}_{n,2}) \delta t, \end{aligned}$$

where ${\textbf {u}}_{n+1}$ is the solution at the next time step. Here, ${\textbf {u}}_{n,1}$, ${\textbf {u}}_{n,2}$ are the intermediate variables required to compute ${\textbf {u}}_{n+1}$, i.e., they are the solutions at two quadrature points between the *n*-th and $(n+1)$-th time step. Hence, there are two nonlinear equations (C5a) and (C5b), in which the non-linearity is determined by ${\textbf {f}}$ and one linear equation (C5c) at each time step.

## Appendix D: Supplement to Sect. “Hyper-elasticity problem in multi-scale framework”

Assume a quasi-static equilibrium PDE:

D7 $$\begin{aligned} \mathrm{div \,}{\textbf {P}} = 0. \end{aligned}$$

The objective is then to find a displacement field ${\varvec{{u}}}$ for a given constitutive law, e.g.,

$$\begin{aligned} {\textbf {P}} = \frac{\partial \mathcal {E}}{\partial {\textbf {F}}} \quad \text {with } \mathcal {E} = \textrm{Tr}({\textbf {F}}^T\cdot {\textbf {F}}) - 2 - 2\ln {(\det {\textbf {F}})} + \lambda (\det {\textbf {F}} - 1)^2, \end{aligned}$$

where $\mathcal {E}$ is the strain energy density, ${\textbf {P}}$ and ${\textbf {F}}$ are the nominal stress and deformation gradient, respectively, and $\lambda$ is the Lamé parameter. The deformation gradient is of the form: ${\textbf {F}} := \mathrm{grad \,}{\varvec{{y}}} = {\textbf {I}} + \mathrm{grad \,}{\varvec{{u}}}$, where ${\varvec{{y}}}$ is the position after deformation.

The micro-scale PDE, implemented in the micro-scale $\Omega _{\textrm{m}}$ that represents a point at $\Omega$ with the micro-structure, reads: For a given macro-scale strain ${\textbf {F}}_{\textrm{M}}$ and Lamé parameter $\lambda$, find micro-scale displacement ${\varvec{{u}}}_{\textrm{m}} \in H^1(\Omega _{\textrm{m}}) \times H^1(\Omega _\textrm{m})$, such that

D8 $$\begin{aligned} \mathrm{div_m \,}{\textbf {P}}_{\textrm{m}} ({\varvec{{u}}}_{\textrm{m}}; \lambda ) = 0, \end{aligned}$$

complemented with the Hill-Mandel condition driven periodic boundary condition for ${\varvec{{u}}}_{\textrm{m}}$ and an anti-periodic boundary condition for ${\textbf {P}}_{\textrm{m}}$:

D9 $$\begin{aligned} {\varvec{{u}}}_{\textrm{m}}^+ - {\varvec{{u}}}_{\textrm{m}}^-&= \varepsilon ({\textbf {F}}_{\textrm{M}} - {\textbf {I}}) \cdot ({\varvec{{x}}}^+ - {\varvec{{x}}}^-); \end{aligned}$$

D10 $$\begin{aligned} {\textbf {P}}_{\textrm{m}}^+ \cdot {\varvec{{n}}}^+&= - {\textbf {P}}_{\textrm{m}}^- \cdot {\varvec{{n}}}^-. \end{aligned}$$

Here, $^+$ and $^-$ denote the opposite sides of the edge of the micro-scale computational domain, $\Omega _{\textrm{m}}$. The periodic effect can be seen in an example in Fig. 17. Note that the computational domain for micro-scale simulation is a square (scaled from its size $\varepsilon$ to unit), $\Omega _{\textrm{m}}$, shown in Fig. 16 in the scope of the considered experiment. We use the notation $\mathrm{grad_m \,}$ and $\mathrm{div_m \,}$ to express the gradient and divergence operator in the domain after scaling. This means that $\mathrm{grad_m \,}:= \varepsilon \, \mathrm{grad \,}$ and $\mathrm{div_m \,}:= \varepsilon \, \mathrm{div \,}$. To solve the above-introduced problem, we introduce an intermediate variable $\tilde{{\varvec{{u}}}}$ such that ${\varvec{{u}}}_{\textrm{m}} = {\varvec{{u}}}_{\textrm{M}} + \varepsilon ({\textbf {F}}_{\textrm{M}} - {\textbf {I}}) \cdot ({\varvec{{x}}} - {\varvec{{x}}}_0) + \tilde{{\varvec{{u}}}}$, where ${\varvec{{u}}}_{\textrm{M}}$ denotes the macro-scale displacement. This decomposition yields ${\textbf {F}}_{\textrm{m}} = {\textbf {F}}_{\textrm{M}} + \mathrm{grad_m \,}\tilde{{\varvec{{u}}}}$, leads to a zero mean condition:

D11 $$\begin{aligned} \int _{\Omega _{\textrm{m}}} \tilde{{\varvec{{u}}}} \, \textrm{d}V = 0. \end{aligned}$$

Using (D9), we have:

D12 $$\begin{aligned} \tilde{{\varvec{{u}}}}^+ - \tilde{{\varvec{{u}}}}^- = 0. \end{aligned}$$

Given conditions (D11)–(D12), we define the trial function space and the test function space as $H(\Omega _{\textrm{m}}) := \{{\varvec{{v}}} \in H^1(\Omega _{\textrm{m}}) \times H^1(\Omega _{\textrm{m}}) | (\int _{\Omega _{\textrm{m}}} {\varvec{{v}}} \, \textrm{d}V = 0) \wedge ({\varvec{{v}}}^+ - {\varvec{{v}}}^- = 0)\}$. Using condition (D10), the weak formulation of (24), for a given ${\textbf {F}}_{\textrm{M}}$, reads as follows: Find $\tilde{{\varvec{{u}}}} \in H(\Omega _{\textrm{m}})$, s.t. for all ${\varvec{{v}}} \in H(\Omega _\textrm{m})$,

D13 $$\begin{aligned} \int _{\Omega _{\textrm{m}}} {\textbf {P}}_{\textrm{m}}(\tilde{{\varvec{{u}}}}; {\textbf {F}}_{\textrm{M}}, \lambda ) : \mathrm{grad_m \,}{\varvec{{v}}} \, \textrm{d}V = 0. \end{aligned}$$

In fact, the left-hand-side of (D13) is the virtual work in the direction ${\varvec{{v}}}$. This is equivalent to the derivative of $\mathcal {E}$ in the aforementioned direction. By defining the microscopic stiffness tensor ${\textbf {K}}_{\textrm{m}} = \frac{\partial {\textbf {P}}_{\textrm{m}}}{\partial {\textbf {F}}_{\textrm{m}}} = \frac{\partial ^2 \mathcal {E}}{\partial {\textbf {F}}_{\textrm{m}}^2}$, the Gâteaux derivative (required by the Newton’s method) of the left-hand-side of (D13) is

$$\begin{aligned} d \, \textrm{LHS} = \int _{\Omega _{\textrm{m}}} \frac{\partial {\textbf {P}}_{\textrm{m}}}{\partial {\textbf {F}}_{\textrm{m}}} \cdot d \, {\textbf {F}}_{\textrm{m}} : \mathrm{grad_m \,}{\varvec{{v}}} \, \textrm{d}V = \int _{\Omega _{\textrm{m}}} {\textbf {K}}_{\textrm{m}} \cdot \mathrm{grad_m \,}d \, \tilde{{\varvec{{u}}}} : \mathrm{grad_m \,}{\varvec{{v}}} \, \textrm{d}V. \end{aligned}$$

Finally, to get ${\textbf {K}}_{\textrm{M}} := {\textbf {K}}_\textrm{eff} = \frac{\partial {\textbf {P}}_{\textrm{M}}}{\partial {\textbf {F}}_\textrm{M}}$, we have

$$\begin{aligned} {\textbf {K}}_{\textrm{M}} = \int _{\Omega _{\textrm{m}}} \frac{\partial {\textbf {P}}_{\textrm{m}}}{\partial {\textbf {F}}_{\textrm{M}}} \, \textrm{d}V {\approx } \sum _{p=1}^{Q^{\textrm{r}}} \frac{\partial \theta _p^{\textrm{RB}}}{\partial {\textbf {F}}_{\textrm{M}}} \int _{\Omega _\textrm{m}} \xi _p^{\textrm{RB}} \, \textrm{d}V. \end{aligned}$$

## Abbreviations

DC: Diverging-converging
DIRK: Diagonally implicit Runge-Kutta method
EIM: Empirical interpolation method
FE(M): Finite element (method)
ML: Machine learning
MOR: Model order reduction
MSE: Mean-square error
NIDL: Non-intrusive deep learning method
NN: Neural network
PDE: Partial differential equation
PDNN: Projection-driven neural network
PINN: Physics-informed neural network
PRNN: Physics-reinforced neural network
POD: Proper orthogonal decomposition
RB(M): Reduced basis (method)
ResNet: Residual neural network
StdLy: Standard layer
TTDN: Two-tier deep network

## Footnotes

1 In a physical model, the derived quantity of interest is obtained by applying the constitutive law to the given parameters and unknown state, and then is verified and updated by checking whether it satisfies the governing equation.

2 Since the system matrix in FEM is generally sparse and banded, it is more efficient to use sparse solvers. Sparse solvers are generally not amenable to complexity analysis because of the sparsity. Generally the complexity for a sparse solver is between $\mathcal {O}((N^\textrm{f})^{3/2})$ and $\mathcal {O}((N^{\textrm{f}})^3)$.

3 The Kolmogorov *N*-width is a criterion to measure the efficiency of *N* basis functions to approximate a non-linear manifold. With the same *N*, the higher the Kolmogorov *N*-width is, the worse the approximation performs. For the mathematical definition, please refer to [5].

4 We do not show the relative error via NIDL for $N^{\textrm{r}} > 7$ because they explode and exceed 1.
